# Effectiveness of digital interventions to reduce school‐age adolescent sexual risks: A systematic review

**DOI:** 10.1111/jnu.13015

**Published:** 2024-08-08

**Authors:** Ana Aguilar‐Quesada, Alba Sierra‐Yagüe, María González‐Cano‐Caballero, José Antonio Zafra‐Egea, Marta Lima‐Serrano

**Affiliations:** ^1^ Hospital Universitario San Cecilio Granada Spain; ^2^ Catalan Institute of Health Barcelona Spain; ^3^ Department of Nursing, Faculty of Health Sciences University of Granada Granada Spain; ^4^ Department of Nursing of the Faculty of Health Sciences of UManresa Fundació Universitària del Bages, University of Vic, Central University of Catalonia Barcelona Spain; ^5^ Department of Nursing, School of Nursing, Physiotherapy, and Podiatry University of Seville Seville Spain; ^6^ Institute of Biomedicine of Seville (IBiS) Sevilla Spain

**Keywords:** adolescent, health education, internet‐based intervention, sexual behavior

## Abstract

**Introduction:**

The increase in risky sexual behaviors among adolescent students has sparked alarm and has become an area of research interest. As adolescents prioritize confidentiality and accessibility, digital interventions are becoming increasingly relevant in sex education. We therefore posed the following research question: Are digital application interventions effective to prevent risky sexual behaviors in school adolescents?

**Design:**

A systematic peer review was conducted between January and December 2023 in five databases (PubMed, Web of Science, Scopus, EMBASE, and PsycINFO) without restricting for language or year of publication.

**Method:**

We included randomized control trials or quasi‐experimental studies that measured the effectiveness of interventions targeting young people aged 10–19 years or their parents and developed in a school setting. Interventions aimed at young people with intellectual disabilities, learning difficulties, or any disease requiring a specific intervention were excluded.

**Results:**

The search ultimately yielded 27 studies covering a total of 18 digital interventions that demonstrated positive effects, not maintained over time, on knowledge, attitudes, and behaviors, although the latter to a lesser extent.

**Discussion:**

We have found very interesting digital interventions with effects, among others, on knowledge, attitudes, and contraceptive use in adolescents. In general, digital interventions have positive effects on knowledge and attitudes, but it is more difficult to modify behaviors with strictly digital interventions or combined with complementary face‐to‐face sessions or group class activities.

**Conclusion:**

We thus believe that digital interventions are adequate to reduce adolescent sexual risk behaviors, and our systematic review facilitates the implementation of these interventions by sharing existing digital interventions that have had positive effects, as well as the main characteristics a digital intervention should possess to reduce sexually risky behaviors in adolescents.

**Clinical relevance:**

Digital interventions with adolescents improve sexual behaviors and can be a valuable resource in education on this topic due to their accessibility and confidentiality, two key points for young people.

## INTRODUCTION

Adolescence is a period of transition that involves great physical, psychological, social, and emotional changes, which vary based on several factors such as the education received, the environment, context, and skills, among others (Simanjuntak et al., 2023), and can affect both sex education and sexual behavior. Increases in risky sexual behaviors among adolescent students—such as having sexual intercourse at younger ages or not using condoms in their sexual intercourses (Inchley et al., 2018)—have sparked alarm and become an area of interest for global public health researchers, who have sought innovative approaches to promote better healthy sexual outcomes (Haruna et al., 2018). Due to the increase in risky behaviors, an increase in sexually transmitted infections (STIs) has been observed in this population (World Health Organization [WHO], 2019). Of the estimated 39.0 million people living with HIV worldwide in 2022, 2.58 million were children aged 0–19. Human papillomavirus (HPV) is the most common STI, and there were about 43 million HPV infections in 2018, many among people in their late teens and early 20s. Chlamydia, gonococcus, and HIV are common STIs as well (WHO, 2021a). In addition to sexually transmitted diseases, adolescent girls face the possibility of unintended pregnancies that may end in abortions, eclampsia, puerperal endometritis, and systemic infections, which can be dangerous for the adolescent health (Sully et al., 2019).

The United Nations has proclaimed sexual and reproductive health to be a universal human right (Panchaud, 2022). According to WHO, sexual health is a state of physical, emotional, mental, and social well‐being in relation to sexuality. Sexual health requires a positive and respectful approach to sexuality and sexual relationships, as well as the possibility of having pleasurable and safe sexual experiences, free of coercion, discrimination, and violence (WHO, 2024). So, the above data show that education on sexuality and reproduction is necessary to equip young people with the knowledge, skills, attitudes, and values they need to determine and enjoy their sexuality, physically and emotionally, individually and in relationships. Traditionally, the best setting for sex education is the school, where health professionals and teachers have easier access to young people (Goldfarb & Lieberman, 2021). However, given that adolescents prioritize confidentiality and accessibility over reliable health information, digital interventions have recently become more relevant for sex education (Brayboy et al., 2018). Digital interventions—also called Internet‐based or web‐based interventions—are the use of the Internet to facilitate the dissemination of health‐related information and to connect patients to support (National Library of Medicine, 2023). This can include text message interventions, smartphone applications, and websites. The use of digital interventions is a promising method to address sexual and reproductive health disparities among different groups of adolescents. Such interventions are also more easily spread and implemented and have higher efficacy and greater engagement compared to traditional behavioral interventions (Brayboy et al., 2018).

The WHO recommends listening to young people to design sustainable digital solutions, because they are experts on their health needs, the technologies they use, and how they access to information (WHO, 2021b). Technologies are part of the routine of adolescents, who use them for information seeking, communication, and leisure. Most (95%) of teens now report having a smartphone or access to one (Robb et al., 2019). These mobile connections are, in turn, motivating more persistent online activities: 45% of teens now say they are online on a near‐constant basis. However, there is no clear consensus about the effect that social networks and online interventions have on the lives of young people today. Most teens describe that effect as mostly positive (31%) or mostly negative (24%), but nearly half (45%) say that effect is neither positive nor negative (Robb et al., 2019). It is thus very important to ensure good digital literacy and to teach young people the necessary resources to use technology for matters of sexual health. A digital intervention in schools could thus be a promising solution to the promotion of sexual health and the prevention of risky sexual behavior among adolescents.

Several literature reviews have been published on digital interventions to prevent risky sexual behavior in adolescents (Brayboy et al., 2018; Goldfarb & Lieberman, 2021; Nourimand et al., 2022), but there has been no standardization of interventions and a clear trend toward the use of technologies (Brayboy et al., 2018) or the use of the school as an intervention setting. These reviews provide extensive support for a broad and comprehensive treatment of sex education, as well as for the effectiveness of digital interventions (Brayboy et al., 2018; Goldfarb & Lieberman, 2021; Nourimand et al., 2022). However, these reviews included articles with different designs, including randomized control trials (RCTs), cross‐sectional studies, protocols, experimental and quasi‐experimental studies, and cohorts that may have influenced the findings (Nourimand et al., 2022). For this reason, new reviews on this subject are recommended to overcome these limitations.

The objective of this systematic review is to summarize and evaluate existing research on digital interventions to improve adolescents' knowledge, attitudes, intention, and behaviors related to sexual risk and how such interventions have been implemented. For future researchers, it is intended to be useful to generate effective online interventions on sexuality in schools. This review investigated the following *research question*: Are digital application interventions effective to prevent risky sexual behaviors in school‐age adolescents?

## METHODS

To achieve the research objectives, a systematic review was carried out between January and December 2023 in accordance with the PRISMA Declaration. This systematic review has been registered with PROSPERO under ID CRD42022306728. PROSPERO is an international database of prospectively registered systematic reviews in health and social care, welfare, public health, education, crime, justice, and international development and is funded by the National Institute for Health Research (NIHR). PROSPERO aims to provide a comprehensive list of systematic reviews registered at inception to help avoid duplication and reduce the opportunity of reporting bias by allowing comparison of the completed review with what was planned in the protocol.

### Eligibility criteria

To be included, articles should be RCTs or quasi‐experimental trials. There were no restrictions on language and year of publication. Articles should include adolescents (10–19 years old) who received digital application interventions, interventions developed in any school, and an evaluation of the effectiveness of the digital intervention. We decided that the digital interventions would be developed at schools to unify as much as possible the characteristics and context of the population and the development and characteristics of the interventions. Our screening team excluded an article if it was a case‐controlled trial, cohort study, case report, case series, non‐original research, secondary report, commentary, editorial, review, a study that did not report outcomes related to sexual prevention, a study weak methodological quality, or a study that included adolescents with intellectual disability, learning disabilities, another disability, risk, or any illness that required a specific intervention.

### Information sources

The search was conducted independently by two researchers in five electronic databases (PubMed, Web of Science, Scopus, EMBASE, and PsycINFO). We also manually searched the reference list of included studies to identify potentially relevant studies.

### Research strategy

To select the keywords, an initial search was carried out in different review papers with a similar subject matter to obtain a better overview of the topic to be addressed and to obtain all the essential information for this review. A specialized librarian was also consulted. The search strategy was elaborated following the population, intervention/exposure, comparator, outcome, time, and study design (PICOTS) structure as visualized in Table 1 and the review question was: “Are digital application interventions effective to prevent risky sexual behaviors in school‐age adolescents?” The following search strategy was used: (Adolescent OR student OR teen OR school‐age OR minor) AND (application OR software OR device OR “mobile application” OR “mobile app” OR m‐health OR e‐health OR “internet based” OR “web based” OR “Computer‐assisted instruction” OR “Educational technology” OR online) AND (“sexual behavior” OR “sexual health” OR “STD” OR “sexually transmitted diseases” OR “sexual and reproductive health” OR “HIV” OR “pregnancy, unwanted” OR “sex offenses”) AND (intervention OR “sex education” OR “health education” OR program OR prevention OR “health promotion”). There were no restrictions on language, year of publication, or article type. The search strategy adapted to the different databases can be found in Table S1.

**TABLE 1:** Population, intervention/exposure, comparator, outcome, time, and study design (PICOTS) criteria.

PICOTS criteria	
Population	Adolescents (10–19‐year‐olds) in school. Any social, economic, and cultural class
Intervention/Exposure	Digital interventions to prevent risky sexual behaviors in adolescents using web, application mobile, online program, etc.
Comparator	Habitual methodology educational intervention to prevent risky affective‐sexual behaviors in adolescents or no intervention
Outcome	Prevention of risky sexual behaviors in adolescents and their measure of effects: condom use, number of postcoital contraceptives, number of unwanted pregnancies, and number of induced abortions and sexually transmitted diseases
Time	Not applicable
Study design	Randomized controlled trials (RCTs) or quasi‐experimental trials

### Selection process and data collection

**FIGURE 1:**
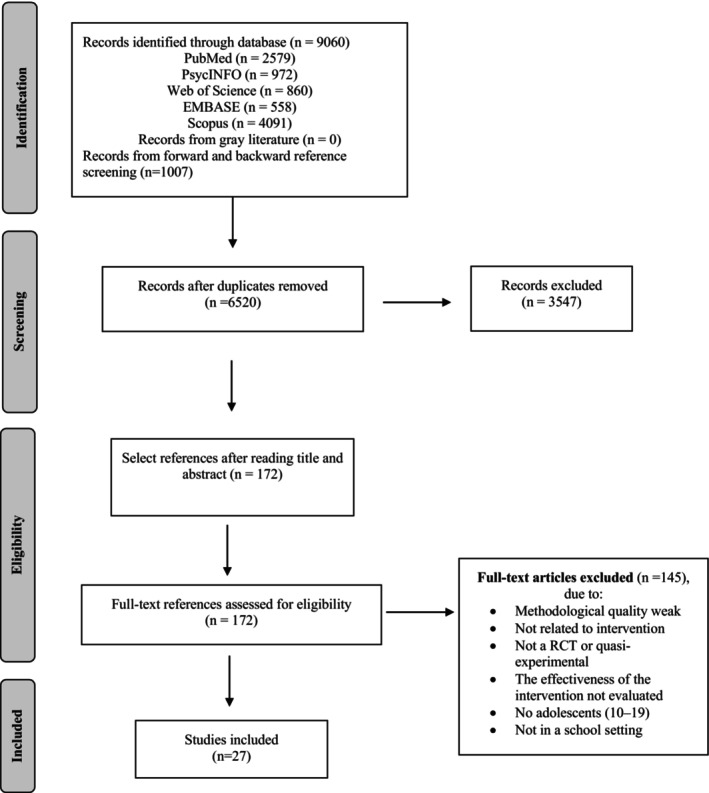
Flowchart for the selection of articles for the systematic review.

All search results were imported into a Google spreadsheet to screen title, author/s, and bibliographic reference. Two reviewers selected studies in two different rounds. First, both reviewers read the title and abstract and sorted by inclusion or exclusion criteria. Duplicate articles were removed manually. Second, the included data articles extracted independently by the two reviewers were imported into an online Google spreadsheet in a standardized order. Both reviewers agreed on the review of the articles and all discrepancies and disagreements were discussed and resolved by reviewing the inclusion or exclusion criteria and reaching a consensus or asking to third reviewer. Finally, the whole process was validated by a senior research member. This screening and data extraction procedure is represented through the PRISMA flow diagram in Figure 1.

### Data items and synthesis methods

Data extracted from the included articles were collected in three standardized tables for team consensus. These tables displayed the information by project, because more than one paper often referred to the same project. The first table showed study characteristics, design, country of study, population, sample, and follow‐up, outcomes included, and main effects of the intervention (only those with *p* < 0.05). When possible, Cohen's *d* was calculated; we considered values of 0.2 low, 0.5 moderate, and 0.8 large effects. When offered, we used the odds ratio (1.50 low effect, 3.50 moderate, and 9.00 large effect). However, given the heterogeneity of the studies with respect to interventions, participants, and outcome measures, we did not calculate pooled effect sizes.

The second table summarized the positive or negative effects of the interventions on primary outcomes (sexual intercourse and contraceptive use) and secondary outcomes (knowledge and attitudes) in a period of less than 6 months and in a period longer than 6 months. The third table captured intervention features such as setting, theory, objective, number, frequency and duration of sessions, facilitators, resources, and implementation. Any missing and unclear information was described as “no data” in the tables.

### Bias assessment

Methodological quality was rated using the Quality Assessment Tool for Quantitative Studies developed by the Effective Public Health Practice Project (EPHHP). The quality assessment items were selection bias, study design, confounders, blinding, data collection methods, and withdrawal/dropout (EPHHP, 2022). Both reviewers were trained in the use of study‐rating instruments. We scored each item with “strong”, “moderate” and “weak” depending on the answers to different questions that describes Quality Assessment Tool for Quantitative Studies Dictionary. Finally, we classified articles as “strong”, “moderate”, and “weak”. A third reviewer reconciled any disagreement between the main researchers.

### Effect measures

The principal outcome was prevention of risky sexual behaviors in adolescents whose measured effects were sexual intercourse and contraceptive use as primary outcomes and knowledge and attitudes as secondary outcomes.

## RESULTS

As can be seen in Figure 1, the literature search in the various databases yielded 9060 records (2579 PubMed, 972 PsycINFO, 860 Web of Science, 558 EMBASE, and 4091 Scopus) and 1007 records from the bibliographies of the articles included. We sorted the identified publications by title and found that there were 3547 duplicates. After the removal of duplicates, the remaining records were filtered in accordance with the inclusion and exclusion criteria by title and abstract. Examining the titles and abstracts resulted in 172 eligible records. The full texts of the remaining records were subsequently examined. Studies that came from the reference lists of the included articles were examined. A total of 27 publications were ultimately considered eligible and retained for the review.

The dates of the publications of the 27 studies included in this systematic review ranged from 1987 to present; however, most were published after 2017. The projects were developed mainly in the United States, with other sites in Puerto Rico (Varas‐Díaz et al., 2019), Mexico (Castillo‐Arcos et al., 2016; Doubova et al., 2017), Brazil (Halpern et al., 2008), China (Hu et al., 2023; Jin et al., 2021), Malaysia (Nik Farid et al., 2018), Korea (Jeong et al., 2017), Uganda (Ybarra et al., 2013, 2015), and Kenya (Halpern et al., 2008). Two of the projects did not define the location of the intervention (Fiellin et al., 2017; Roberto, Zimmerman, Carlyle, Abner, Cupp, et al., 2007).

### Methodological features

The methodological features of the studies are shown in Table S2.

#### Study design

Regarding the study design, all were cluster RCTs except for five articles, which were quasi‐experimental (Castillo‐Arcos et al., 2016; Halpern et al., 2008; Jeong et al., 2017; Kann, 1987; Roberto, Zimmerman, Carlyle, & Abner, 2007), and one field trial (Doubova et al., 2017). Most of the publications worked with a control group and an intervention group, except for three publications that used two experimental groups and one control group (Markham et al., 2012; Murry et al., 2018, 2019), one publication that used an experimental group and two control groups (Kann, 1987), and one publication that worked with two experimental groups and two control groups (Halpern et al., 2008).

Finally, seven studies used a pretest and posttest measure (Castillo‐Arcos et al., 2016; Halpern et al., 2008; Nik Farid et al., 2018; Roberto, Zimmerman, Carlyle, Abner, Cupp, et al., 2007; Scull et al., 2018; Tortolero et al., 2010; Widman et al., 2020), one study used a pretest and 3‐month‐follow‐up measures (Raghupathy et al., 2013), another study used a pretest and 10‐week‐follow‐up measure (Roberto, Zimmerman, Carlyle, & Abner, 2007), two studies used a pretest and 6‐month‐follow‐up (Murry et al., 2018, 2019), and one study used a pretest and 12‐month‐follow‐up measure (Peskin et al., 2015) without an immediate posttest. The rest of the studies used more than two measures of effects.

#### Quality assessment

Of the 27 articles included in the systematic review, 13 were classified as “strong” in methodological quality and 14 as “moderate”. Four publications (Chong et al., 2013; Haruna et al., 2019, 2021; Lightfoot et al., 2007), that were originally included in the systematic review because they met the inclusion criteria, were removed from the review because their methodological quality was “weak”, and the research team decided to exclude it to obtain higher quality results.

#### Participants

Most of the articles included did not provide complete information about their participants, which made it difficult to draw a clear demographic profile. In the 27 included articles, the participants were adolescents and in three of them parents were also included (Murry et al., 2018, 2019; Varas‐Díaz et al., 2019).

The participants were mostly enrolled in schools where digital interventions were carried out. Other settings included an urban educational institution (Castillo‐Arcos et al., 2016), a community organization (Varas‐Díaz et al., 2019), and urban after‐school and summer camp programs (Fiellin et al., 2017). The studies whose sample were the parents of adolescents were also developed in schools (Murry et al., 2018, 2019; Varas‐Díaz et al., 2019).

Regarding gender, most of the interventions were aimed at both boys and girls. However, there was a greater tendency for girls to participate. However, we found four studies in which the proportion of boys in the study population was higher (Fiellin et al., 2017; Nik Farid et al., 2018; Ybarra et al., 2013, 2015) and two studies with an equal proportion of boys and girls (Jin et al., 2021; Scull et al., 2018). Another study had two study groups in which one group (Brazil) had a higher proportion of girls and the other group (Kenya) had a higher proportion of boys (Halpern et al., 2008). There was one study that did not describe the gender of its participants (Potter et al., 2016).

Most studies calculated the mean age and standard deviation of their sample, fluctuating between 12.09/0.79 (Jin et al., 2021) and 16.25/0.76 (Widman et al., 2020), except for the study whose intervention was developed by parents whose mean age and standard deviation was 42.5/8.25 (Varas‐Díaz et al., 2019). Three studies did not make this calculation and included their sample in age groups, from 12 years old (Kann, 1987; Nik Farid et al., 2018) to 19 years old (Kann, 1987). There were two studies that did not provide this information (Jeong et al., 2017; Potter et al., 2016).

When taken together, the size of the samples of the 27 studies ranged from 88 (Jeong et al., 2017) to 3244 (Potter et al., 2016) participants, with a mean of 751.071 participants (SD = 786.979).

The retention rate of the interventions included in the systematic review was above 70%, except in two studies (Roberto, Zimmerman, Carlyle, & Abner, 2007; Roberto, Zimmerman, Carlyle, Abner, Cupp, et al., 2007) where these data were not described, another study where the retention rate was 69.40% (Tortolero et al., 2010), and another study which divided its sample into two groups, one of which achieved a retention rate of 77% (Brazil) and the other 51% (Kenya) (Halpern et al., 2008).

#### Intervention characteristics

##### Intervention goal

Table S3 summarizes the characteristics of the interventions obtained in the systematic review. From the 27 publications included, 18 digital interventions were extracted. All digital interventions in our systematic review sought to improve adolescent sexual health. Most of them achieved this goal by increasing sexual knowledge, increasing contraceptive use, and improving adolescent sexual attitudes. Three interventions aimed to improve adolescent sexual health by delaying sexual initiation (Markham et al., 2012; Peskin et al., 2015, 2019; Potter et al., 2016; Tortolero et al., 2010), one intervention aimed to improve media literacy education to improve adolescent sexual health (Scull et al., 2018, 2022), and two interventions aimed to improve adolescent communication with parents (Murry et al., 2018, 2019) or interpersonal communication (Kann, 1987).

##### Intervention facilitators

All but three interventions (Halpern et al., 2008;Nik Farid et al., 2018; Widman et al., 2018, 2020) had facilitators who developed the intervention and helped the adolescents carry it out. These facilitators were either schoolteachers with prior training, members of the research team, a member of the health care team, or technology intervention assistants or facilitator trainers specifically chosen for the intervention.

##### Intervention strategies

Most of the interventions were digital, but there were three studies that used website and face‐to‐face sessions in the school (Castillo‐Arcos et al., 2016; Doubova et al., 2017; Jeong et al., 2017), and four studies used computer‐based activities and group‐based classroom activities (Markham et al., 2012; Peskin et al., 2019; Potter et al., 2016; Tortolero et al., 2010).

The methodologies used in the different interventions were mainly videos (Hu et al., 2023; Jin et al., 2021; Nik Farid et al., 2018; Peskin et al., 2015; Raghupathy et al., 2013; Varas‐Díaz et al., 2019; Widman et al., 2018, 2020; Ybarra et al., 2013, 2015), virtual role‐playing (Doubova et al., 2017; Fiellin et al., 2017; Murry et al., 2018, 2019; Peskin et al., 2015; Raghupathy et al., 2013), games (Doubova et al., 2017; Fiellin et al., 2017; Widman et al., 2018, 2020), or quizzes (Peskin et al., 2015; Raghupathy et al., 2013; Widman et al., 2018, 2020). Five studies did not provide details about which methodology was used in their interventions (Castillo‐Arcos et al., 2016; Halpern et al., 2008; Kann, 1987; Murry et al., 2018, 2019; Scull et al., 2018, 2022).

##### Theorical framework

Most interventions were based on theoretical models of behavior change—that is, social cognitive theory (Markham et al., 2012; Peskin et al., 2019; Potter et al., 2016; Tortolero et al., 2010; Varas‐Díaz et al., 2019), theory of planned behavior (Scull et al., 2018, 2022; Varas‐Díaz et al., 2019), theories of reasoned action and planned behavior (Scull et al., 2018, 2022; Varas‐Díaz et al., 2019; Widman et al., 2018, 2020), or the information‐motivation‐behavioral skills model (Peskin et al., 2015; Ybarra et al., 2013, 2015) among others. Seven studies did not describe the theoretical model of their intervention theoretical model (Fiellin et al., 2017; Halpern et al., 2008; Hu et al., 2023; Jin et al., 2021; Kann, 1987; Nik Farid et al., 2018; Raghupathy et al., 2013).

##### Intervention sessions

There was a mean of 8.51 digital sessions in the intervention across studies (SD = 7.12) and a mode is six sessions (Castillo‐Arcos et al., 2016; Halpern et al., 2008; Murry et al., 2018, 2019; Raghupathy et al., 2013; Varas‐Díaz et al., 2019; Ybarra et al., 2013, 2015). The shortest interventions had 1 digital session (Nik Farid et al., 2018; Widman et al., 2018, 2020) and the longest intervention has 24 digital sessions (Markham et al., 2012; Peskin et al., 2019; Potter et al., 2016; Tortolero et al., 2010).

The duration of the sessions of the interventions ranged between 15 and 90 min, with a mean of 49.21 (SD = 19.58). There was one study that did not say how long the digital sessions lasted, and this was omitted from the calculation of the mean (Halpern et al., 2008).

##### Intervention contents

The contents of the sessions of the different interventions are shown in Table S3. Main contents of sessions were knowledge about HIV/STI, pregnancy and contraception, sexual skills and strategies to improve communication.

### Main effects

Significant effects (*p* < 0.05) are shown in Table 2 and Table S2. All studies showed some positive effect on adolescent sexual health, either in terms of sexual knowledge, sexual attitude, or sexual behavior such as contraceptive use or healthier sexual intercourse compared to receiving no sexual intervention or receiving the usual sexual intervention from their school. Most interventions that aimed to improve sexual health education in adolescents found low to moderate effects. There were four publications that found large effects for secondary outcomes, with *d* = 4.78 in HIV/sexually transmitted disease (STD) knowledge (Widman et al., 2018); *d* = 1.27 in HIV/STD knowledge (Widman et al., 2020), *d* = 1.16 in sexual knowledge (Hu et al., 2023), *d* = 1.38 in STI knowledge (Jeong et al., 2017); and *d* = 0.90 in sexual knowledge (Roberto et al., 2008).

**TABLE 2:** Outcomes effects.

Intervention	Author/s and year	Primary outcome				Secondary outcome			
		Sexual intercourse		Contraceptive use		Knowledge		Attitudes	
		<6 months	≥6 months	<6 months	≥6 months	<6 months	≥6 months	<6 months	≥6 months
“You and Me”	Hu et al. (2023)	NS	NS	NS	NS	+	+	+	+
	Jin et al. (2021)	ND	ND	ND	ND	+	+↓	+	−
“Media Aware”	Scull et al. (2022)	ND	ND	ND	ND	+	ND	ND	ND
	Scull et al. (2018)	ND	ND	+	ND	ND	ND	ND	ND
“HEART”	Widman et al. (2020)	ND	ND	+	ND	+	ND	+	ND
	Widman et al. (2018)	ND	ND	+	+	+	+	+	+
“Cuídalos”	Varas‐Díaz et al. (2019)	ND	ND	ND	ND	ND	ND	ND	ND
“Pathways for African American Success” (PAAS)	Murry et al., 2019	ND	ND	ND	ND	ND	ND	ND	ND
	Murry et al. (2018)	ND	ND	ND	ND	ND	ND	ND	ND
“Malaysian Care for Adolescent Project” (MyCAP)	Nik Farid et al. (2018)	ND	ND	ND	ND	+	ND	NS	ND
“It's Your Game” (IYG)	Peskin et al. (2019)	+	NS	+	NS	+	+	NS	NS
	Potter et al. (2016)	NS	−	NS	NS	+	+	NS	NS
	Tortolero et al. (2010)	+	+	+	NS	+	+	ND	ND
“IYG‐Tech”	Peskin et al. (2015)	NS	NS	+	+	+	+	ND	ND
Smartphone app	Jeong et al. (2017)	ND	ND	+	+	+	+	ND	ND
Internet‐based educational intervention	Doubova et al. (2017)	ND	ND	NS	+	+	+	+	+
“PlayForward”	Fiellin et al. (2017)	NS	NS	ND	ND	+	+	+	+
“Connect”	Castillo‐Arcos et al. (2016)	ND	ND	ND	ND	+	ND	ND	ND
“CyberSenga”	Ybarra et al. (2015)	ND	ND	+	+	+	+	+	+
	Ybarra et al. (2013)	+	NS	+	NS	ND	ND	ND	ND
“Abstinence and Contraception Education Storehouse”: ACES	Raghupathy et al. (2013)	+	ND	ND	ND	+	ND	ND	ND
“Risk avoidance” (RA) and “Risk reduction” (RR)	Markham et al. (2012)	+	+	+	+	+	+	ND	ND
“Teen Web”	Halpern et al. (2008)	ND	ND	−	ND	−	ND	ND	ND
Computer‐ and Internet‐based intervention	Roberto et al. (2008)	+	ND	+	ND	+	ND	+	ND
	Roberto, Zimmerman, Carlyle, and Abner (2007)	+	ND	NS	ND	+	ND	+	ND
	Roberto, Zimmerman, Carlyle, and Abner (2007)	+	ND	NS	ND	+	ND	+	ND
“CAI”	Kann (1987)	ND	ND	ND	ND	+	ND	+	ND

Abbrevitions: ND, no data; NS, not significant; +, positive significant effects; −, negative significant effects.

Focusing on the evaluation time framework, from studies that assess primary sexual outcomes, eight studies showed positive effects on sexual intercourse (Markham et al., 2012; Peskin et al., 2019; Raghupathy et al., 2013; Roberto et al., 2008; Roberto, Zimmerman, Carlyle, & Abner, 2007; Roberto, Zimmerman, Carlyle, Abner, Cupp, et al., 2007; Tortolero et al., 2010; Ybarra et al., 2013), of which only two had such effects at 6 months (Markham et al., 2012; Tortolero et al., 2010). These effects at short time were low, ranging from *d* = 0.11 (Roberto, Zimmerman, Carlyle, & Abner, 2007) to *d* = 0.3 (Raghupathy et al., 2013).

Regarding conceptive use, 11 studies (Jeong et al., 2017; Markham et al., 2012; Peskin et al., 2015, 2019; Roberto et al., 2008; Scull et al., 2018; Tortolero et al., 2010; Widman et al., 2018, 2020; Ybarra et al., 2013, 2015) demonstrated low positive effects with Cohen's *d* between *d* = 0.20 (Roberto et al., 2008) and *d* = 0.43 (Widman et al., 2018). Five studies maintained these effects over time up to 6 months (Jeong et al., 2017; Markham et al., 2012; Peskin et al., 2015; Widman et al., 2018; Ybarra et al., 2015). Additionally, one study did not show significant data before 6 months but showed positive effects at 6 months (Doubova et al., 2017). Finally, and one study showed negative effects on contraceptive use (Halpern et al., 2008).

Regarding secondary outcomes, 21 studies found that sexual knowledge was improved by the digital intervention (Castillo‐Arcos et al., 2016; Doubova et al., 2017; Fiellin et al., 2017; Hu et al., 2023; Jeong et al., 2017; Jin et al., 2021; Kann, 1987; Markham et al., 2012; Nik Farid et al., 2018; Peskin et al., 2015, 2019; Potter et al., 2016; Raghupathy et al., 2013; Roberto et al., 2008; Roberto, Zimmerman, Carlyle, & Abner, 2007; Roberto, Zimmerman, Carlyle, Abner, Cupp, et al., 2007; Scull et al., 2022; Tortolero et al., 2010; Widman et al., 2018, 2020; Ybarra et al., 2015), with 11 of these studies maintaining the improvement over time (Doubova et al., 2017; Fiellin et al., 2017; Hu et al., 2023; Jeong et al., 2017; Markham et al., 2012; Peskin et al., 2015, 2019; Potter et al., 2016; Tortolero et al., 2010; Widman et al., 2018; Ybarra et al., 2015). A study showed that the level of sexual knowledge dropped at 6 months (Jin et al., 2021). These positive effects were divided into seven studies that had a low effect with Cohen's *d* between *d* = 0.13 (Roberto, Zimmerman, Carlyle, Abner, Cupp, et al., 2007) and *d* = 0.39 (Castillo‐Arcos et al., 2016), one study with moderate effects (*d =* 0.61; Roberto, Zimmerman, Carlyle, & Abner, 2007) and five studies with large effects on knowledge whose Cohen's *d* was between *d* = 0.90 (Roberto et al., 2008) and *d* = 4.78 (Widman et al., 2018). The remaining studies did not give a Cohen's *d*.

Regarding sexual attitudes, 7 eleven demonstrated positive effects (Doubova et al., 2017; Fiellin et al., 2016; Hu et al., 2023; Jin et al., 2021; Kann, 1987; Roberto et al., 2008; Roberto, Zimmerman, Carlyle, & Abner, 2007; Roberto, Zimmerman, Carlyle, Abner, Cupp, et al., 2007; Widman et al., 2018, 2020; Ybarra et al., 2015). These effects were, in general, moderate with Cohen's *d* between *d* = 0.55 (Widman et al., 2020) and *d* = 0.78 (Widman et al., 2018). They were maintained over time, except for one article in which the positive effects decreased (Jin et al., 2021) and another in which long‐term effects were not described (Widman et al., 2020).

#### Intervention strategy

The interventions that were exclusively digital showed a greater increase in sexual knowledge (Hu et al., 2023; Jin et al., 2021; Kann, 1987; Nik Farid et al., 2018; Peskin et al., 2015; Raghupathy et al., 2013; Roberto, Zimmerman, Carlyle, & Abner, 2007; Roberto, Zimmerman, Carlyle, Abner, Cupp, et al., 2007; Widman et al., 2018, 2020; Ybarra et al., 2013, 2015), sexual attitude (Halpern et al., 2008; Hu et al., 2023; Jin et al., 2021; Kann, 1987; Raghupathy et al., 2013; Roberto et al., 2008; Roberto, Zimmerman, Carlyle, & Abner, 2007; Roberto, Zimmerman, Carlyle, Abner, Cupp, et al., 2007; Widman et al., 2018, 2020; Ybarra et al., 2013, 2015), sexual communication (Widman et al., 2018, 2020), and self‐efficacy/efficacy (Halpern et al., 2008; Peskin et al., 2015; Roberto et al., 2008; Roberto, Zimmerman, Carlyle, & Abner, 2007; Roberto, Zimmerman, Carlyle, Abner, Cupp, et al., 2007). However, sexual behavior was not modified by the intervention, except in one case (Kann, 1987). The articles that measured results over a period of 6 months showed these results were not sustained over time (Hu et al., 2023; Jin et al., 2021; Raghupathy et al., 2013; Widman et al., 2018, 2020).

Five interventions combined face‐to‐face sessions with digital sessions (Castillo‐Arcos et al., 2016; Doubova et al., 2017; Jeong et al., 2017; Markham et al., 2012; Peskin et al., 2019; Potter et al., 2016; Tortolero et al., 2010) and their effects were generally positive. In less than 6 months, there was an improvement in sexual knowledge (Castillo‐Arcos et al., 2016; Doubova et al., 2017; Jeong et al., 2017; Peskin et al., 2019; Potter et al., 2016; Tortolero et al., 2010), self‐efficacy (Jeong et al., 2017; Peskin et al., 2019; Potter et al., 2016; Tortolero et al., 2010), sexual attitude (Doubova et al., 2017; Markham et al., 2012), and contraceptive use (Doubova et al., 2017; Jeong et al., 2017; Markham et al., 2012; Peskin et al., 2019; Potter et al., 2016; Tortolero et al., 2010), and in more than 6 months, there were positive effects in contraceptive use (Jeong et al., 2017; Markham et al., 2012), sexual knowledge (Jeong et al., 2017; Markham et al., 2012; Peskin et al., 2019; Potter et al., 2016), and sexual intercourse (Markham et al., 2012; Tortolero et al., 2010). Better results were obtained if the intervention was completed (Doubova et al., 2017).

#### Participants and exposure

Digital interventions that focused on adolescents but also on their parents (Murry et al., 2018, 2019; Varas‐Díaz et al., 2019) showed improved communication between them and, therefore, in the sexual health of the adolescents. There were also differences between those adolescents who received the full digital intervention (“full exposure”: 13 lessons) and those who did not receive the full exposure (“no exposure”: 0 lessons, “low exposure”: 1–4 lessons, “mid‐exposure”: 5–8 lessons and “high exposure”: 9–12 lessons), with the finding that “full exposure” and “mid‐exposure” students were less likely than “low exposure” students to initiate sex (Peskin et al., 2015). Better results were also seen in those interventions that included booster sessions as opposed to those interventions that only provided the main sessions (Roberto et al., 2008; Roberto, Zimmerman, Carlyle, & Abner, 2007; Roberto, Zimmerman, Carlyle, Abner, Cupp, et al., 2007; Ybarra et al., 2013, 2015). One digital intervention focused on improving adolescent media literacy succeeded in increasing sexual knowledge and media and sexual health, but did not influence communication (Scull et al., 2018, 2022). Another digital intervention was a video game that improved sexual knowledge and attitudes, but these improvements were not sustained over time, nor did they change participants' sexual behaviors (Fiellin et al., 2017).

## DISCUSSION

This systematic review summarizes existing digital interventions to reduce sexual risk behaviors and thus improve adolescents' sexual health. We have found very interesting digital interventions with effects, among others, on knowledge, attitudes, and contraceptive use in adolescents. In general, digital interventions have positive effects on knowledge and attitudes, but it is more difficult to modify behaviors with strictly digital interventions or combined with complementary face‐to‐face sessions or group class activities (Castillo‐Arcos et al., 2016; Doubova et al., 2017; Jeong et al., 2017; Markham et al., 2012; Peskin et al., 2019; Potter et al., 2016; Tortolero et al., 2010). For example, dynamics such as group activities, face‐to‐face role‐playing, and small group discussions have been shown to improve the behavioral change outcomes of individuals (Herbert & Lohrmann, 2011). However, there is also evidence showing positive effects on behaviors with all‐digital interventions (Downs et al., 2004; Noar et al., 2009). Therefore, we can deduce that one of the most important points for sexual education is the dedication of time and resources to adolescents and their parents. These digital interventions can also work to improve the literacy of adolescents in the media so that they can seek accurate and appropriate information on their own (Scull et al., 2018, 2022). Another important finding of our review is the effects are not maintained over time as much as we would like (Hu et al., 2023; Jin et al., 2021), because they begin to diminish with the passage of months. The beneficial effect of reinforcement sessions is noteworthy (Roberto et al., 2008; Roberto, Zimmerman, Carlyle, & Abner, 2007; Roberto, Zimmerman, Carlyle, Abner, Cupp, et al., 2007; Ybarra et al., 2013, 2015).

Regarding the target population of the interventions, most work only with adolescents, but we found two interventions that also include parents because they were based on the protective factor of families in adolescent health. Including parents in this type of intervention means parents become more aware of their children's sexual problems and improve communication with them. This improved communication leads to greater knowledge about STIs and how to prevent them, improved self‐efficacy and increased intention in health behaviors, but to a lesser extent (Murry et al., 2018, 2019; Varas‐Díaz et al., 2019).

Most interventions were based on theoretical models of behavior change, recognizing that the adoption of this approach produces better results (Jackson & Waters, 2005; Peters et al., 2009).

Adherence to the interventions was evaluated with the retention rate of the different studies, which is generally high (above 70%); this suggests that the digital interventions were well accepted by adolescents as they break the dynamics of lectures and classes on sexual health and make the information more interesting for the students. It also guaranteed the confidentiality and autonomy they demanded (Brayboy et al., 2018).

Regarding applicability, although most of the interventions were carried out with a sufficient sample, but only one study developed its intervention in two different countries with different contexts to obtain different results, starting with adherence to the intervention by adolescents, thus demonstrating that the context influences the different interventions or participant characteristics (Halpern et al., 2008).

The strength of this systematic review is that it worked with five databases in addition to a previous and subsequent reference search of all the articles to add new possible studies to the review without language, publication date, or other restrictions and with a focus on RCTs and quasi‐experimental studies to increase the quality of the results. Only interventions conducted within schools were considered for inclusion. The methodological quality of the included studies ranged from high to moderate. It is possible that some relevant studies may have inadvertently been omitted, despite our careful selection of comprehensive keywords.

However, the heterogeneity in terms of differences in interventions, study designs, methodologies, and variables used for outcome measures prevents us from providing a comprehensive synthesis of the results, because we could not always calculate the effect size or other measures that would facilitate the objective comparison of one intervention with another. It was difficult to establish a specific time for the short‐ and long‐term effects, because each intervention had its follow‐up tests at different times (e.g., 1 month, 6 months, 1 year), so we have described the results by differentiating the effects in a period of less than 6 months or in a period longer than 6 months. The characteristics and size of the population also differed from one study to another, as did the primary and secondary results. Nevertheless, we were able to unify criteria to obtain clearer results.

Another limitation found in this systematic review was that the digital interventions to improve participants' sexual behaviors were controlled by the researchers and facilitators, who were with the adolescents while they received the intervention, resolving doubts and reminding them of the sessions. The interventions were also mostly financially compensated and conducted in settings known and safe for participants, which likely increased adherence. We therefore propose to investigate these digital interventions to improve adolescent sexual behaviors in a less controlled manner to more realistically capture how adolescents engage in these types of interventions.

The studies included in this systematic review also reported outcomes related to different eHealth interventions (e.g., mobile, web‐based, and apps), and the joint evaluation of these different modalities may affect final judgments. Future studies could examine the preventive impact of a single eHealth intervention on sexually risky behaviors based on the results of our study. Another limitation is there is little research on long‐term changes in adolescent sexual behaviors: most are short‐term. We therefore suggest that the same projects be carried out again but measuring long‐term results to verify that these changes are maintained over time or if, on the contrary, it is necessary to change the intervention strategy or introduce booster sessions.

This review has practical implications as its findings can assist future researchers, educators, healthcare professionals, and policymakers in developing suitable health education programs for young people. We outline the ideal characteristics for the development of a digital intervention to reduce adolescent sexual behaviors, and we also provide information on existing digital interventions that have proven effective in achieving the common goal of improving sexual health in these young individuals.

## CONCLUSIONS

In conclusion, our systematic review highlights the positive impact of digital interventions on adolescent sexual health, demonstrating significant improvements in knowledge, attitudes, and behavior. While these interventions contribute to positive changes across these dimensions, our findings suggest that the effects may not be sustained over time. This emphasizes the importance of incorporating reinforcement sessions as a critical component in the design and implementation of these interventions to ensure prolonged effectiveness.

Furthermore, our review underscores the versatility of these interventions, which can be effectively directed not only at adolescents but also at their parents. Recognizing the influential roles parents play in shaping adolescent sexual health outcomes, interventions that involve both parties have shown positive results, particularly in enhancing knowledge about STDs, improving self‐efficacy, and fostering positive intentions toward health behaviors, albeit to a lesser extent.

It is worth noting that exclusive digital interventions exhibit favorable outcomes in promoting adolescent sexual health. However, our findings also indicate that interventions combining digital approaches with group or face‐to‐face interactions are equally effective. This implies that the selection of an intervention strategy to address adolescent sexual behaviors should be context‐specific, considering the unique characteristics and dynamics of the target population.

In summary, the success of digital interventions in improving adolescent sexual health is evident, but the challenge lies in sustaining these positive effects over time. By incorporating reinforcement sessions and tailoring interventions to the specific context of the population, stakeholders, including researchers, educators, healthcare professionals, and policymakers, can enhance the overall effectiveness of interventions aimed at reducing adolescent sexual risk behaviors. This comprehensive approach acknowledges the multifaceted nature of adolescent sexual health and underscores the importance of adapting strategies to ensure lasting positive outcomes.

### CLINICAL RESOURCES

WHO Sexual health: https://www.who.int/health‐topics/sexual‐health#tab=tab_2.

WHO Sexually transmitted infections (STIs): https://www.who.int/health‐topics/sexually‐transmitted‐infections#tab=tab_1.

American Sexual Health Association: https://www.ashasexualhealth.org.

Medline Teen sexual Health: https://medlineplus.gov/teensexualhealth.html.

It's your game…Keep it real: https://www.etr.org/ebi/programs/its‐your‐game/.

## CONFLICT OF INTEREST STATEMENT

The authors declare that they have no conflicts of interest with respect to the research, authorship, and/or publication of this article.

## Supporting information


Table S1.
Table S2.
Table S3.

## Data Availability

The data that support the findings of this study are available on request from the corresponding author. The data are not publicly available due to privacy or ethical restrictions.
