# Supplementary material for: Effectiveness of digital interventions to reduce school‐age adolescent sexual risks: A systematic review
Source: J Nurs Scholarsh. 2024 Aug 8;57(2):342–53. doi: 10.1111/jnu.13015 (PMC11931981; doi:10.1111/jnu.13015)
Supplement: Supplementary file 1 — Table S1. Table S2. Table S3. [file JNU-57-342-s001.docx]

**Table S1. *Database search strategy (Pubmed, Psycinfo, Web of Science, EMBASE, Scopus)***

| **Database** | **Descriptors** | **Limits** |
| --- | --- | --- |
| **Pubmed** | ((((Adolescent OR student OR teen OR school-age OR minor)) AND (application[Title/Abstract] OR software[Title/Abstract] OR device[Title/Abstract] OR "mobile application"[Title/Abstract] OR "mobile app"[Title/Abstract] OR m-health[Title/Abstract] OR e-health[Title/Abstract] OR "internet based"[Title/Abstract] OR "web based"[Title/Abstract] OR "Computer-assisted instruction"[Title/Abstract] OR "Educational technology"[Title/Abstract] OR online[Title/Abstract])) AND (("sexual behavior" OR "sexual health" OR "STD" OR "sexually transmitted diseases" OR "sexual and reproductive health" OR "HIV" OR "pregnancy, unwanted" OR "sex offences"))) AND ((intervention[Title/Abstract] OR "sex education"[Title/Abstract] OR "health education"[Title/Abstract] OR program[Title/Abstract] OR prevention[Title/Abstract] OR "health promotion"[Title/Abstract])) | Article types: unfiltered  Publication year: unfiltered  Languages: unfiltered |
| **Psycinfo** | (Adolescent OR student OR teen OR school-age OR minor ) AND tiab( application OR software OR device OR "mobile application" OR "mobile app" OR m-health OR e-health OR "internet based" OR "web based" OR "Computer-assisted instruction" OR "Educational technology" OR online) AND tiab("sexual behavior" OR "sexual health" OR "STD" OR "sexually transmitted diseases" OR "sexual and reproductive health" OR "HIV" OR "pregnancy, unwanted" OR "sex offences") AND tiab(intervention OR "sex education" OR "health education" OR program OR prevention OR "health promotion") NOT tiab("men who have sex with men") | Article types: unfiltered  Publication year: unfiltered  Languages: unfiltered |
| **Web of Science** | ((((AB=(Adolescent OR student OR teen OR school-age OR minor)) AND AB=(application OR software OR device OR "mobile application" OR "mobile app" OR m-health OR e-health OR "internet based" OR "web based" OR "Computer-assisted instruction" OR "Educational technology" OR online)) AND AB=("sexual behavior" OR "sexual health" OR "STD" OR "sexually transmitted diseases" OR "sexual and reproductive health" OR "HIV" OR "pregnancy, unwanted" OR "sex offences")) AND AB=(intervention OR "sex education" OR "health education" OR program OR prevention OR "health promotion")) NOT AB=("men who have sex with men" OR MSM) | Article types: unfiltered  Publication year: unfiltered  Languages: unfiltered |
| **EMBASE** | (adolescent:ab,ti OR student:ab,ti OR teen:ab,ti OR 'school age':ab,ti OR minor:ab,ti) AND (application:ab,ti OR software:ab,ti OR device:ab,ti OR 'mobile application':ab,ti OR 'mobile app':ab,ti OR 'm health':ab,ti OR 'e health':ab,ti OR 'internet based':ab,ti OR 'web based':ab,ti OR 'computer-assisted instruction':ab,ti OR 'educational technology':ab,ti OR online:ab,ti) AND ('sexual behavior':ab,ti OR 'sexual health':ab,ti OR 'std':ab,ti OR 'sexually transmitted diseases':ab,ti OR 'sexual and reproductive health':ab,ti OR 'hiv':ab,ti OR 'pregnancy, unwanted':ab,ti OR 'sex offences':ab,ti) AND (intervention:ab,ti OR 'sex education':ab,ti OR 'health education':ab,ti OR program:ab,ti OR prevention:ab,ti OR 'health promotion':ab,ti) NOT ('men who have sex with men':ab,ti OR msm:ab,ti) | Article types: unfiltered  Publication year: unfiltered  Languages: unfiltered |
| **Scopus** | ( TITLE-ABS-KEY ( adolescent OR student OR teen OR school-age OR minor ) AND TITLE-ABS-KEY ( application OR software OR device OR "mobile application" OR "mobile app" OR m-health OR e-health OR "internet based" OR "web based" OR "Computer-assisted instruction" OR "Educational technology" OR online ) AND TITLE-ABS-KEY ( "sexual behavior" OR "sexual health" OR "STD" OR "sexually transmitted diseases" OR "sexual and reproductive health" OR "HIV" OR "pregnancy, unwanted" OR "sex offences" ) AND TITLE-ABS-KEY ( intervention OR "sex education" OR "health education" OR program OR prevention OR "health promotion" ) AND NOT TITLE-ABS-KEY ( "men who have sex with men") ) | Article types: unfiltered  Publication year: unfiltered  Languages: unfiltered |

**Table S2. *Results***

| **Intervention** | **Author/s** | **Study characteristics, Design; Country; Population; Sample & follow-up; Retention rate; % gender; Age; Outcomes included** | **Main effects** | **Risk of bias** |
| --- | --- | --- | --- | --- |
| **“You and Me”** Internet-based sexuality education program | Hu et al., 2023 | CRCT, EG & CG; China; 10º grader; 29 schools.  Pretest, immediate posttest and 12-month-follow-up: 1528 (EG), 1268 (CG); 88.73%; 47.44%♀, 52.56%♂; Mean age/SD (EG): 16.13/0.83. Mean age/SD (CG): 16.04/0.85; sexual knowledge, sexual attitude, symptoms of sexually transmitted infections, pregnancy, contraceptive use at last sexual intercourse, condom use at last same-sex sexual behavior | Multivariate logistic regression analysis showed significant improvements on sexual knowledge and sexual attitude at short term (d=1.16, 95% CI=1.08–1.24 and d=0.61, 95% CI=0.53–0.69 respectively) and long term (d=0.38, 95% CI=0.31–0.46 and d=0.23, 95% CI=0.16–0.31 respectively). On behavioral outcomes, no effects were found. | Moderate |
|  | Jin et al., 2021 | CRCT, EG & CG, design: China; 7º grader; 6 schools. Pretest, immediate posttest and 12-month-follow-up: 241 (EG), 187 (CG); 81.44%; 50%♀, 50%♂ (EG), 52.32%♀, 47.68♂ (CG); Mean age/SD (EG): 12.9/0.79, Mean age/SD (CG): 12.7/0.87; sexuality knowledge and attitude | The GLM model showed positive effects on sexual knowledge at posttest (β=4.65, 95% CI=4.12–5.17, p<0.001) and 12-month-follow-up (β=2.39, 95% CI=1.85–2.93, p<0.001) and on attitudes at posttest (β=1.25, 95% CI=1.00–1.50, p<0.001) and at 12-month-follow-up (β=0.49, 95% CI=0.23–0.75, p<0.0002). Positive effects of the intervention on attitudes were observed in all aspects at the first follow-up test, sustained effects were only observed at the second follow-up on attitudes toward discussing sex (β=0.14, 95% CI=0.06–0.22) and seeking help (β=0.17, 95% CI=0.07–0.26). The effects of the intervention on attitudes toward gender equity, gender-based violence, and homosexuality were no longer statistically significant. | Moderate |
| **“Media Aware”** Mobile health (mHealth), media literacy education program | Scull et al., 2022 | CRCT, EG & CG; United States; 9º, 10º graders; 17 schools. Pretest, immediate posttest and 3-month-follow-up: 291 (EG), 74 (CG); 74.88%; 52.70%♀, 42.99%♂, 4.32% self-described; Mean age/SD: 14.42/0.70; critical thinking about media messages, sexual health (norms, knowledge, and intention to use protection) and communication (norms, efficacy, intention and communication with other people) | Multilevel regression analysis showed at posttest an effect perceived realism of media messages (M/SD EG=2.15/0.05, M/SD CG=2.00/0.04, d=0.23, p=0.02), media message deconstruction skills (M/SD EG=4.66/0.45, M/SD CG=3.38/0.46, d=0.17, p=0.05), sexual health knowledge (M/SD EG=11.24/0.12, M/SD CG=10.74/0.16, d=0.26, p=0.01), risky teen sex descriptive norms (M/SD EG=31.47/1.59, M/SD CG=36.70/2.11, d=0.19, p=0.05). There was a significant interaction of gender and condition for acceptance of dating violence (b=-0.19, p<0.05) At posttest, the effectiveness was found for boys in the EG , who reported less acceptance of dating violence (M/SD=1.44/0.05) than boys in the CG (M/SD=1.60/0.07; d=0.29). The same effect was not present among girls (d=-0.06). There was a significant interaction of gender and condition for descriptive normative beliefs about teen sex (b=8.06, p<0.05). At posttest, girls in the EG reported lower estimates of sex amongst their peers (M/SD=32.06/1.69) than girls in the CG (M/SD=40.44/2.44; d=0.38) Finally, there was a significant interaction of gender and condition for sexual health communication with a parent (b=-0.36, p<0.05). At posttest, girls in the EG reported more frequent sexual health communication with a parent (M/SD=2.17/0.08) than girls in the CG (M/SD=1.80/0.12, d=0.37). | Moderate |
|  | Scull et al., 2018 | CRCT, EG & CG; United States; 7º, 8º graders; 9 schools. Pretest and immediately posttest: 625 (EG), 301 (CG); 105.22%; 49%♀, 51%♂; Mean age: 13.02 (EG), 12.84 (CG); Primary sexual health outcomes: intentions to engage in the following behaviors: have sex, use contraception/protection, communicate with a partner about sexual health, communicate with a parent or another trusted about sexual health and communicate with a doctor about sexual health. Secondary sexual health outcomes: attitudes, normative beliefs, and self-efficacy related to teen sexual activity and sexual refusal, contraception/protection use, and sexual health communication with partners, parents or another trusted adult, and a doctor or another medical professional. Media-related outcomes: media skepticism, perceived similarity, perceived realism, deconstruction skills | Multilevel models showed effects on the intentions to use contraception (M/SD EG=3.44/0.62, M/SD CG=3.37/0.71, d=0.17, p=0.012), and increased intentions to communicate with a doctor or other medical professional (M/SD EC=3.50/0.64, M/SD CG=3.30/0.77, d=0.2, p=0.002) and a romantic partner (M/SD EG=3.45/0.64, M/SD CG=3.38/0.66, d=0.17, p=0.012) should they decide to have sex compared to the control group. Intervention students reported more self-efficacy for contraception use (M/SD EG=3.03/0.63, M/SD CG=2.64/0.83, d=0.31, p<0.001) and communicating with a romantic partner (M/SD EG=3.19/0.60, M/SD CG=3.20/0.63, d=0.19, p=0.003) at posttest compared with the control group. Analyses suggest that changes in deconstruction skills mediated the program’s impact on change in intentions to communicate with a doctor or other medical professional; *Media Aware* led to increases in media deconstruction (b=1.24 p<0.001) and, in turn, increases in media deconstruction led to increases in intent to communicate with a doctor/medical professional (b=0.44, p<0.001). The mediation effect estimate was 0.546 (95% CI=0.261–0.886). However, the mediation analyses were conducted with data from one time point, and, therefore, the direction of causality cannot be guaranteed. | Strong |
| **“HEART”** Interactive online program | Widman et al, 2020 | CRCT, EG & CG (Growing Minds: to cultivate academic and social growth mindsets); United States; 10º, 11º graders; 1 school. Pretest and immediately posttest: 113 (EG), 113 (CG); 91.60%; 58%♀, 42%♂; Mean age/SD: 16.25/0.76; communication intentions, condom intentions, HIV/STD knowledge, condom attitudes, condom norms, self-efficacy, sexual assertiveness | Linear regression analysis showed effects of the program on sexual communication intentions (M/SD EG=4.43/0.83, M/SD CG=3.97/1.15, d=0.57, p<0.001), condom use intentions (M/SD EG=4.38/1.15, M/SD CG=3.96/1.43, d=0.24, p=0.020), HIV/STD knowledge (M/SD EG=7.68/1.79, M/SD CG=5.74/1.86, d=1.27, p<0.001), condom attitudes (M/SD EG=3.85/0.96, M/SD CG=3.22/0.94, d=0.55, p<0.001), condom norms (M/SD EG=3.74/1.03, M/SD=3.28/1.13, d=0.41, p=0.001), safer sex self-efficacy (M/SD EG=3.30/0.66, M/SD CG=3.21/0.56, d=0.23, p=0.031) and sexual assertiveness compared to control participants (M/SD EG=3.88/0.98, M/SD CG=3.68/0.75, d=0.29, p=0.023). | Moderate |
|  | Widman et al., 2018 | CRCT, EG & CG (Growing Minds: to cultivate academic and social growth mindsets); United States; 10º grader; 4 schools. Pretest, immediately posttest and 4-month-follow-up: 107 (EG), 115 (CG); 95.05%; 100%♀; Mean age:15.2; communication intentions, communication self-efficacy, sexual assertiveness self-report, HIV and STD knowledge, condom attitudes, condom norms, condom self-efficacy, condom intentions. | Linear generalized estimating equation models showed effects of the program on communication intentions (M/SD EG=87.0/22.5, M/SD CG=78.5/27.9, d=0.33, p<0.001), sexual assertiveness self-report (M/SD EG=3.6/0.8, M/SD CG=3.1/0.8, d=0.60, p<0.001), HIV and STD knowledge (M/SD EG=10.1/1.5, M/SD CG=7.0/2.0, d=4.78, p<0.001), condom attitudes (M/SD EG=4.1/0.8, M/SD CG=3.5/0.7, d=0.78, p<0.001), condom norms (M/SD EG=3.9/0.9, M/SD CG=3.6/1.0, d=0.35, p=0.003), condom self-efficacy (M/SD EG=2.8/0.8, M/SD CG=2.3/0.8, d=0.43, p<0.001)  Girls who completed *HEART* had statistically significantly greater knowledge regarding HIV and other STDs (M/SD EG=8.2/2.0, M/SD CG=7.4/2.0, d=0.56, p<0.001), more positive condom attitudes (M/SD EG=4.1/0.8, M/SD CG=3.7/0.8, d=0.45, p<0.001) and greater condom self-efficacy (M/SD EG=2.7/0.9, M/SD CG=2.5/0.8, d=0.30, p=0.007) at 4 months compared with girls in the CG. | Strong |
| **“Cuídalos”** Web-based intervention | Varas-Díaz et al., 2019 | CRCT, EG & CG (physical activity program); Puerto Rico; 18 community organizations and 38 public schools. Pretest and 3,6,12-month-follow-up: 330 parent/adolescent dyads (EG), 330 parent/adolescent dyads (CG). 51.82%♀, 47.88%♂ (adolescents); Age (adolescents): 13-17; 89.55%♀, 10.15%♂ (parents); 70.30%; Mean age/SD (parents): 42.5/8.25; general parent-adolescent communication in general and about: sexual risk, sexual prevention, sexual protection, sexual peer pressure, comfort and stigma.  mediators: sexual communication attitudes, subjective norms, self-efficacy, sexual communication intentions | Generalized estimating equation models were conducted to study the main effect (group by time interaction) on the outcome (Model-M1), on mediators (M2), mediators on the outcomes (M3) and the effect of the intervention on outcomes controlled by the mediators (M4). Focus on the effect of the intervention (M1), parents who participated in the EG reported more sexual peer pressure communication (mean difference=0.02, standard error=0.01, p=0.039), more sexual prevention communication (mean difference=0.02, standard error=0.01, p=0.031), more sexual protection communication (mean difference=0.02, standard error=0.01, p=0.008), and more sexual risk communication (mean difference=0.02, standard error=0.01, p=0.010) per month than parents in the control group. Mediation analyses demonstrated that self-efficacy, sexual communication attitudes, and subjective norms fully mediated the effect of the intervention on sexual peer pressure communication and sexual prevention communication, and partially mediated the effect of the intervention on sexual protection communication and sexual risk communication. | Strong |
| **“Pathways for African American Success” (PAAS)** Technology-delivered [HIV risk](https://www--sciencedirect--com.us.debiblio.com/topics/medicine-and-dentistry/human-immunodeficiency-virus-risk-behavior) prevention program | Murry et al., 2019; Murry et al., 2018 | CRCT, 2EG (in-person facilitator-led PAAS small group condition and self-directed PAAS technology condition) & CG (with home-mailed educational materials condition); United States; 6º grader; 5 schools. Pretest and 6-month-follow-up: 101 (EG1 Facilitator), 124 (EG2 PASS technology) and 109 (CG); 81,07%; 54%♀, 46%♂ (adolescents); Mean age (adolescents): 11.4; 84.3%♀, 15.7%♂ (parents); Mean age (parents): 40; parent and youth targeted outcomes (articulated norms/expectations about risk engagement, open, supportive, family communication, frequency of conversations, discussion quality, conflicted ineffective communication), youth targeted outcomes (risk engagement intentions) | Compared with caregivers assigned to the CG, significant positive intervention unadjusted effects were observed for caregivers assigned to both the small group condition (M/SD=10.64/2.55, d=0.39, 95% CI=0.11–0.66) and the technology condition (M/SD=10.31/2.53, d=0.26, 95% CI=0.00–0.52) on the Frequency of Conversations subscale. In addition, significant positive intervention effects were observed for those assigned to the small group condition on the Open, Supportive Family Communication composite scale (M/SD=38.09/5.40, d=0.31, 95% CI=0.02–0.61) and the Discussion Quality subscale (M/SD=13.04/2.99, d=0.30, 95% CI=0.01–0.59). A significant positive treatment effect was observed for those assigned to the technology condition on the Articulated Norms and Expectations about Risk Engagement scale (M/SD=41.58/4.74, d=0.29, 95% CI=0.03–0.55).  Compared with youth assigned to the control condition, those assigned to the small group condition reported significantly higher scores on the Articulated Norms and Expectations about Risk Engagement scale (M/SD=40.30/7.09, d=0.27, 95% CI=0.00–0.55). Moreover, youth assigned to the PAAS technology arm reported significantly lower Risk Engagement Intentions ((M/SD=8.47/1.31, d=-0.36, 95% CI=-0.63−-0.10) than their counterparts assigned to the in-person, facilitator-led group condition. All p<0.05 significant differences in youth's performance as a function of group assignment to these two delivery formats emerged. | Moderate |
| **“Malaysian Care for Adolescent Project” (MyCAP)**  Website for online SRH education | Nik Farid et al., 2018 | CRCT, EG & CG; Malaysia; 1 school. Pretest and immediately posttest: EG (101), CG (108); 100%; 46,6%♀, 53,8%♂; Age: 12; sexual and reproductive health knowledge, sexual and reproductive attitudes. | The study found a statistically significant effect in the mean knowledge score (M/SD EG=17.47/3.69, M/SD CG=16.61/3.66, d=0.23, 95% CI=-0.04–0.50, p<0.005). There was no interaction between time and condition. | Strong |
| ***“*It's Your Game” (IYG)** Computer program**.** | Peskin et al., 2019 | CRCT, EG & CG; United States; 7º,8º grader; 10 schools. Pretest and 12, 24-month-follow-up: 804 (EG), 739 (CG); 75.35%; 54.7%♀, 45.3%♂; Mean age/SD: 12,99/0,57; sexual initiation (vaginal or oral sex; vaginal sex only; oral sex only), other sexual risk behaviors among sexually active youth in the last 3 months (had sex without a condom; had sex without an effective contraceptive method; number of times had sex; number of sexual partners; number of times used drugs or alcohol before having sex). used an effective contraceptive method at last intercourse.  *Psychosocial*: knowledge, normative beliefs, perceived self-efficacy (refraining from sex, negotiating condom use with a partner, to obtain and correctly use condoms), personal boundaries, environmental factors, intentions, exploratory outcomes (heard about HPV, heard about HPV shots/vaccines, ever received HPV shots/vaccines, perception of alcohol use of best friends) | A multilevel regression analysis found that there were no significant differences in primary or secondary sexual behavior outcomes between study conditions at the 24-month-follow-up. On psychosocial outcomes in 8º and 9º grader, significant difference was found for knowledge variables: general knowledge about condoms (d=0.29, p<0.001, d=0,22, p<0.001 respectively); general knowledge about HIV/STIs (d=0.28, p<0.001, d=0.36, p<0.001 respectively); knowledge of signs and symptoms of STIs (d=0.31, p<0.001, d=0.015, p=0.007 respectively); exploratory outcomes (heard about HPV AOR=2.22, p<0.001, AOR=1.44, p=0.005 respectively); heard about HPV injections/vaccines (AOR=1.45, p=0.01, AOR=1.33, p=0.04). For the following variables it was statistically significant only in 8º grader: Normative beliefs (to have waited to have sex as an adult (d=0.14, p=0.02), how many other teens their age are having sex (d=0.16, p=0.003); perceived self-efficacy: negotiating condom use with a partner (d=-0.11, p=0.047), to obtain and correctly use condoms (d=0.26, p<0.001); personal limits knowing how far I would go sexually and being able to communicate this to a partner (d=0.11, p=0.04], intentions: having sex in the next year if I have the chance [d=-0.13, p=0.04), using an effective contraceptive method if having sex in the next year (d=0.14, p=0.02). | Strong |
|  | Potter et al., 2016 | CRCT, EG & CG; United States; 7º-9º graders; 24 school. Pretest, posttest and 6, 12, 18-month-follow-up: 1775 (EG), 1469 (CG). 79.13%; Gender: not described; Mean Age: not described; behavioral (initiation of vaginal sex, had vaginal sex in past 3 months). Psychosocial: general beliefs about waiting to have sex or not having sex, condom knowledge, perceived friends’ beliefs about sex and condoms, general knowledge about condoms and STI, perceived self-efficacy about sex and use of condoms, personal limits about communication, intentions to have sex and exposure to risky situations | Multilevel logistic regression analysis showed effect on the program for psychosocial variables in 8th and 9th grade but not in behavioral outcomes. In 8º grader, it was statistically significant for the variables number of reasons to not have sex (b=0.32, 95% CI=0.08–0.56, d=0.10, p<0.05), general condom knowledge (b=13.06. 95% CI=9.97–16.15, d=0.33, p<0.001), general HIV/STI knowledge (b=9.23, 95% CI=6.56–11.89, d=0.28, p<0.001), knowledge of STI signs and symptoms (b=3.67, 95% CI=0.85–6.49, d=0.11, p<0.05), obtain and correctly use condoms (b=0.23, 95% CI=0.14–0.32, d=0.21, p<0.001), know how far I would go sexually and be able to communicate this to partner (b=0.29, 95% CI=0.19–0.40, d=0.22, p<0.001), I know what I think about condom use and can communicate it to my partner (b=0.12, 95% CI=0.04–0.19, d=0.13, p<.01). In 9º grader general condom knowledge (b=5.72, 95% CI=1.24–10.20, d=0.11, p<0.05), general HIV/STI knowledge (b=4.68, 95% CI=1.38–7.98, d=0.12, p <0.05), obtain and correctly use condoms (b=0.13, 95% CI=0.06–0.19, d=0.16, p<0.001), know how far I would go sexually and be able to communicate this to partner (b=0.18, 95% CI=0.08–0.28), d=0.15, p<0.001). | Strong |
|  | Tortolero et al., 2010 | CRCT, EG & CG; United States; 7º, 8º, 9º graders; 10 middle schools. Pretest and posttest: 349 (EG), 558 (CG); 69.40%; 59.1%♀, 40.9%♂; Mean age/SD: 13/0.54; *Primary outcomes:* initiation of sexual activity (any sex, oral sex, vaginal sex and anal sex). *Secondary outcomes*: Psychosocial: beliefs about abstinence, about waiting to have sex until marriage, friends’ beliefs about abstinence, perceptions of friends’ behavior, reasons against having sex, refusal self-efficacy, condom knowledge, beliefs, negotiation self-efficacy and use self-efficacy, global character, exposure to risky situations, STI signs/symptoms, STI knowledge, parental communication, future orientation, intentions to engage in oral sex in the next year, in vaginal sex in the next year, in anal sex in the next year, intentions to remain abstinent until end of high school, abstinent until marriage, intentions to use a condom in the next 3 months, personal limits, perceived norms: most teens wish they waited and most teens my age having sex | A multivariate analysis controlling for confounders showed a program effect on sexual behavior. At 9-month-follow-up EG were less likely to initiate sex than CG (CG=29.9%, EG=23.4%, ARR=1.29, 95% CI=1.02–1.64). This was also significant in Hispanics vs. African-American (CG=27.8%, EG=17.4%, ARR=1.64, 95% CI=1.09–2.47) and female vs. male (CG=26.1%, EG=18.5%, ARR=1.42, 95% CI=1.01–2.01, p<0.05). For the different types of sex, an effect was observed in oral sex in general (CG=17.6%, EG=10.0%, ARR=1.76, 95% CI=1.21–2.56, p<0.01), in African Americans (CG=17.7%, EG=9.5%, ARR=1.84, 95% CI=1.04–3.25, p<0.05) and females (CG=12.8%, EG=5.5%, ARR=2.14, 95% CI=1.12–4.09, p<0.05). For vaginal sex an effect was observed in Hispanics (CG=24.1%, EG=4.8%, ARR=1.67, 95% CI=1.06–2.62, p<0.05), and for anal sex in general (CG=9.9%, EG=3.7%, ARR=2.67, 95% CI=1.45–4.94, p<0.01), in African American race (CG=11.9%, EG=3.3, ARR=3.12, 95% CI=1.21–8.06, p<0.05), males (CG=16.3%, EG=7.5%, ARR=2.31, 95% CI=1.13–4.72, p<0.05), and females (CG=5.8%, EG=1.5%, ARR=3.90, 95% CI=1.16–13.13, p<0.05). And the effect in the last 3 months for sexual intercourse was significant for the variable number of times had sex in the last 3 months: 2 or more vs. 1 in vaginal sex (ARR=1.30, 95% CI=1.02–1.66, p<0.05).  An effect on the beliefs about abstinence until marriage was found at 8º grader (M/SD EG=2.78/0.70, M/SD CG=2.63/0.74, diff adj. mean=0.17, d=0.21, p<0.01) and 9º grader (M/SD EG=2.75/0.69), M/SD CG=2.65/0.73, diff adj. mean=0.12, d=0.14, p<0.01), perceived friends’ beliefs about waiting to have sex at 8º grader (M/SD EG=2.47/0.71, M/SD CG=2.35/0.69, diff adj. mean=0.17, d=0.17, p<0.01), perceived friend’s sexual behavior at 9º grader (M/SD EG=1.77/0.74, M/SD CG=1.83/0.69, diff adj. mean=-0.09, d=-0.0839, p<0.01), self-efficacy to refuse sex at 8º grader (M/SD EG=3.07/0.85, M/SD CG=2.97/0.86, diff adj. mean=0.11, d=0.12, p<0.01), condom knowledge at 8º grader (M/SD EG=2.58/0.71, M/SD CG=2.04/1.01, diff adj. mean=0.53, d=0,62, p<0.01) and 9º grader (M/SD EG=2.41/0.79, M/SD CG=2.25/0.95, diff adj. mean=0.16, d=0,18, p<0.01), perceived friends’ beliefs about condom at 9º grader (M/SD EG=3.32/0.64, M/SD CG=3.21/0.68, diff adj. mean=0.12, d=0.17, p<0.01), self-efficacy to use condoms 8º grader (M/SD EG=2.51/0.39, M/SD CG=2.37/0.44, diff adj. mean=0.12, d=0.12, p<0.01), exposure to risky situations at 8th grade (M/SD EG=0.75/0.65, M/SD CG=0.82/SD=0.69, diff adj. mean=-0.10, d=-0.1044, p<0.05) and 9º grader (M/SD EG=0.86/0.86, M/SD CG=0.96/0.91, diff adj. mean=-0.12, d=-0.113, p<0.05), STI signs/sex knowledge at 8º grader (M/SD EG=0.83/0.24, M/SD CG=0.78/0.26, diff adj. mean=0.10, d=0.20, p<0.01) and 9º grader (M/SD EG=0.82/0.18, M/SD CG=0.76/0.20, diff adj. mean=0.05, d=0.32, p<0.01), HIV/STI knowledge at 8º grader (M/SD EG=0.82/0.21, M/SD CG=0.65/0.29, diff adj. mean=0.17, d=0.67, p<0.01) and 9º grader (M/SD EG=0.80/0.24, M/SD CG=0.70/0.29, diff adj. mean=0.10, d=0.38, p<0.01), reasons not to have sex at 8º grader (M/SD EG=4.87/2.45, M/SD CG=4.29/2.49, diff adj. mean=0.71, d=0.23, p<0.01), intention oral in next year at 8º grader (M/SD EG=1.97/1.26, M/SD CG=2.14/1.24, diff adj. mean=-0.24, d=-0.14, p<0.01), intention abstinent thru high school at 8º grader (M/SD EG=3.16/1.44, M/SD CG=2.89/1.41, diff adj. mean=0.31, d= 0.19, p<0.01). | Strong |
| ***“*It's Your Game (IYG)-Tech”**  (Computer program) | Peskin et al., 2015 | CRCT, EG & CG; United States; 8º grader; 19 schools. Pretest and 12-month-follow-up: 768 (EG), 606 (CG); 89.24%; 59%♀, 41%♂; Mean age/SD: 14.3/0.59; *Primary outcomes:* initiation of sexual activity (any sex, oral sex, vaginal sex, and anal sex). *Secondary outcomes:* Psychosocial: beliefs about abstinence, about waiting to have sex until marriage, friends’ beliefs about abstinence, perceptions of friends’ behavior, reasons against having sex, refusal self-efficacy, condom knowledge, beliefs, negotiation self-efficacy and use self-efficacy, global character, exposure to risky situations, STI signs/symptoms, STI knowledge, parental communication, future orientation, intentions to engage in oral sex in the next year, in vaginal sex in the next year, in anal sex in the next year, intentions to remain abstinent until end of high school, abstinent until marriage, intentions to use a condom in the next 3 months, personal limits, perceived norms: most teens wish they waited and most teens my age having sex | There was no difference in primary outcomes. Significant adjusted differences in beliefs about waiting to have sex until marriage (b=0.08, SE=0.03, 95% CI=0.01–0.14, p<0.05), friends' beliefs about abstinence (b=0.09, SE=0.04, 95% CI=0.01–0.16, p<0.05), condom knowledge (b=0.07, SE=0.01, 95% CI=0.05­–0.10, p<0.01), condom use self-efficacy (b=0.09, SE=0.03, 95% CI=0.02–0.16, p<0.01), STI knowledge (b=0.05, SE=0.02 95% CI=0.01–0.10, p<0.05), perceived norms: most teens wish they waited (b=0.09, SE=0.04, 95% CI=0.00–0.17, p<0.05), perceived norms: most teens my age having sex (b=-0.08, SE 0.04, 95%CI=-0.16–-0.01, p<0.05).  A post hoc analysis was conducted for the association between level of exposure to the intervention and changes in sexual initiation outcomes. The program was effectiveness for or any sex at medium exposure OR=0.42 (p<0.01) and at full exposure OR=0.19 (p<0.01), for vaginal sex at medium exposure OR=0.42 (p<0.05) and full exposure OR=0.20 (p<0.01) and for oral sex at full exposure OR=0.14 (p<0.05) vs low exposure. There was significant difference between the high exposure and low exposure groups. No effect on delay of sexual debut or behavior was found. | Moderate |
| **Smartphone app** | Jeong et al., 2017 | Quasi-experimental non-equivalent control-group time-series, EG & CG (educational booklet);South Korea; 3º grader; 2 high schools. Pretest, immediate posttest and 5-week-follow-up: 47 (EG), 41 (CG); 73.05%; 55.3%♀, 44.7%♂ (EG), 39%♀, 61%♂ (CG); Mean age: no data; STI knowledge, STI vulnerability, STI prevention self-efficacy, STI prevention intentions | Repeated-measures ANOVA showed as significant difference in STI knowledge between the two groups (pretest: M/SD EG=11.79/6.10, M/SD CG=12.17/5.93, d=-0.63, 95% CI=-0.48–0.35, posttest 1: M/SD EG=22.68/4.16, M/SD CG=20.95/5.22, d=0.35, 95% CI=-0.07–0.78, posttest 2: M/SD EG= 22.72/3.59, M/SD CG=16.83/4.92, d=1.38, 95% CI=0.92–1.85, p<0.001 for group, time and time x group). *Smartphone application* condition showed better scores for STI vulnerability (pretest: M/SD EG=11.47/2.01, M/SD CG=11.66/1.84, d=-0.1, 95% CI=-0.52–0.32, posttest 1: M/SD EG=12.87/2.01, M/SD CG=11.88/2.04, d=0.49, 95% CI=0.06–0.91, posttest 2: M/SD EG=12.83/1.75, M/SD CG=11.98/2.02, d=0.452, 95% CI=0.028–0.88, p<0.033 for time and time x group) and for STI prevention self-efficacy (pretest: M/SD EG=8.96/2.18, M/SD CG=8.95/2.32, d=0.00, 95% CI=-0.41–0.42, posttest 1: M/SD EG= 12.74/1.96, M/SD CG=11.71/1.86, d=0.54, 95% CI=0.11–0.96, posttest 2: M/SD EG=12.40/1.64, M/SD CG=10.27/1.83, d=1.22, 95% CI=0.78–1.69, p<0.001 for group, time and time x group). A significant difference in STI prevention intentions was found between the groups (pretest: M/SD EG=11.98/2.51, M/SD CG=11.46/1.98, d=0.23, 95% CI=-0.19–0.65, posttest 1: M/SD EG=13.38/2.12, M/SD CG=12.73/1.61, d=0.34, 95% CI=-0.08–0.76, posttest 2: M/SD EG=13.64/2.06, M/SD CG=11.78/1.51, d=1.02. 95% CI=0.57–1.46, p=0.003 for group, time and group x time) | Moderate |
| **Website** | Doubova et al, 2017 | Field trial, EG & CG (sex education according to the curriculum of the Secretary of Public Education); Mexico; 3º year of secondary school; 2 schools. Pretest, first-month posttest and 4-month-follow-up: 244 (EG), 202 (CG); 97.80%; 52.8%♀, 47.2%♂ (EG), 57.6%♀, 43.3%♂ (CG); Age: 14-15; knowledge of sexually transmitted infections**, attitudes regarding condom use** (reliability and effectiveness, pleasure and stigma associated with condom, embarrassment related to the negotiation and use of a condom and the purchase of a condom), **self-efficacy toward consistent condom use** (certainty about the use of a condom every time when having vaginal sex, when his/her partner has been drinking or using drugs, when he/she has been drinking or using drugs, when his/her partner has been very sexually excited, when he/she is very sexually excited, when his/her partner does not like using a condom, certainty to postpone sex when not having a condom) | Adjusted analysis of Diff-in-Diff was done. At first months it was found an effect of the intervention on **Knowledge of sexually transmitted infections** (β=9.12, p<0.0001).  At fourth months, it was found an effect of intervention on Knowledge of sexually transmitted infections (β=30.34, p<0.0001), reliability and effectiveness of condoms (β=0.65, p<0.0001), pleasure associated with condom use (β=0.66, p<0.00001), stigma attached to being a condom user (β=0.47, p<0.0001), embarrassment related to the negotiation and use of a condom (β=0.75, p<0.0001) and embarrassment related to the purchase of a condom (β=0.59, p<0.001). Moreover there were an effect on certainty about the use of a condom every time when having vaginal sex (β=0.64, p<0.001),  certainty to always use a condom for vaginal sex when his/her partner has been drinking or using drugs (β=1.70, p<0.0001), he/she has been drinking or using drugs (β=1.28, p<0.001), His/her partner has been very sexually excited (β=1.26, p<0.001), he/she is very sexually excited (β=1.62, p<0.0001) and when his/her partner does not like using a condom (β=1.67, p<0.0001). | Moderate |
| **“PlayForward”** Video game | Fiellin et al., 2017 | CRCT, EG & CG; no data; 12 after-schools, schools and summer community programs. Pretest, 6-week-follow-up and 3,6,12-month-follow-up: 166 (EG), 167 (CG); 80.37%; 46.8%♀, 53.2%♂; Mean age/SD: 12,9/1.1; delayed initiation of sexual intercourse; attitudes and knowledge | An intent-to-treat analyses was carried out adjusted for age and gender, it was no found effect on the delayed initiation of sexual intercourse.  Longitudinal mixed-effect models were carried out for secondary outcomes, it was found a statistically significant effect on sexual health attitudes over 12 months (Least squares (LS) mean difference=0.37, 95% CI=0.01–0.72, p=0.04). On sex and age difference, improvement was observed in boys (LS means difference=0.67, p=0.008) but not in girls (LS means difference=0.06, p=0.81) and in the 11-12 years age group (LS means difference=0.71, 95% CI=0.21–1.20, p=0.005). For knowledge, the intervention was effective over 12 months (LS means difference=1.13, 95% CI=0.64–1.61, p<0.001). For girls and boy of EG demonstrated an increase in sexual health knowledge vs. CG (girls: overall LS means difference=1.16, 95% CI=0.46–1.86, p=0.001; boys: LS means difference=1.10, 95% CI=0.43–1.77, p=0.001). For group (age 11-12 years and age 13-14 years) of EG demonstrated an increase in sexual health knowledge vs. CG (younger: LS means difference=1.18, 95% CI=0.50–1.85, p=0.001; older: LS means difference=1.08, 95% CI=0.39–1.78, p=0.002). During the 12-month-follow-up period, there was no difference in intentions to delay sexual debut between the two groups (mean difference=0.10, 95 % CI -0.23–0.43, p=0.56). | Strong |
| **Internet-based intervention** | Castillo-Arcos et al., 2016 | Quasi-experimental with single-stage cluster sampling, EG & CG (video about general health); Mexico; 1 urban educational institution. Pretest and posttest: 96 (EG), 97 (CG); 100%; 61.9%♀, 38.1%♂ (EG), 64.6%♀, 35.4%♂ (CG); Mean age/SD (EG): 15.8/0.5, Mean age/ SD (CG): 15.7 /0.6; sexual resilience, risky sexual behavior | Adjusted multivariate linear regression showed an effect on sexual resilience (β=5.70, p=0.034, 95% CI=0.45–10.96).  In the bivariate analysis, for the intervention condition, the study found an improvement in risk factors as sexual uncertainty (M/SD EG=15.46/5.33, M/SD CG=13.41/4.71, d=0.40, 95% CI=0.12–0.69) and Jalowiec coping (M/SD EG=24.96/13.84, M/SD CG=18.95/16.32, d=0.39, 95% CI=0.11–0.68) and in protective factors as HIV/AIDS and STI knowledge (M/SD EG=27.02/9.02, M/SD CG=23.81/7.53, d=0.39, 95% CI=0.10–0.67), self-esteem (M/SD EG=7.86/5.92, M/SD CG=6.74/6.57, d=0.17, 95% CI=-0.10–0.46), Jalowiec coping (M/SD EG=60.33/27.31, M/SD CG=46.02/34.01, d=0.46, 95% CI=0.18–0.75). | Strong |
| **“CyberSenga”** Healthy sexuality program | Ybarra et al., 2015 | CRCT, EG & CG (traditional sexual education), design: Uganda; 9º, 10º, 11º graders; 4 schools. Pretest and 3, 6-month-follow-up: 366 (EG+CG); 93%; 16%♀, 84%♂; Mean age/SD: 16.1/1.4; HIV prevention related information, attitudes toward: abstinence, condom use, subjective norms for: abstinence, condom use, behavioral intentions for: abstinence, condom use, behavioral skills for: abstinence, condom use | Multilevel growth curve adjusted analysis showed that participants’ HIV prevention-related information changed over time differently depending on experimental condition (B=2.63, p<0.001), attitudes toward condom use (b=0.12, p=0.006), subjective norms for condom use (b=0.12, p=0.015) and behavioral intentions for condom use (b=0.13, p=0.001).  Time by condition by vaginal sex prior to baseline changed by attitudes toward condom use (b=-0.17, p=0.01), subjective norms for condom use (b=-0.25, p=0.001) and behavioral intentions for condom use (b=-0.19, p=0.003). Attitudes toward abstinence (b=0.05, p=0.012), attitudes toward condom use (b=0.22, p<0.001), behavioral intentions for abstinence (b=0.07, p=0.004) and behavioral intentions for condom use (b=0.17, p<0.001) had effects on behavioral skills for condom use. Subjective norms for abstinence (b=0.20, p<0.001) and behavioral intentions for abstinence (b=0.21, p<0.001) had effects on behavioral skills for abstinence. | Strong |
|  | Ybarra et al., 2013 | CRCT, EG & CG, design: Uganda; 4 secondary schools. Pretest and 3, 6-month-follow-up: 183 (EG), 183 (CG); 91.53%; 16.1%♀, 83.9%♂; Mean age/SD: 16.1/1.4; abstinence at six-month follow-up, unprotected vaginal sex at six-month follow-up, abstinence at three-month-follow-up, unprotected sex at three-month-follow-up, trends for the booster group at six-month-follow-up | Adjusted analysis showed at three-month follow-up among abstinent youth at baseline there were more abstinent intervention participants than control participants (EG=88.0% vs. CG=77.3%, AOR=2.27, 95% CI=1.17–4.39, p≤0.05). | Strong |
| **“Abstinence and Contraception Education Storehous”: ACES**  Digital library | Raghupathy et al., 2013 | CRCT, EG & CG (habitual sex education); United States; 1 secondary school and a planned parenthood service. Pretest and 3-month-follow-up: 128 (EG), 122 (CG); 95.31%; 58.9%♀, 42.1%♂; Mean age: 15.5; knowledge, intend to not have sex in next 12 months, number of times had sexual intercourse in past four weeks (all sexually initiated), number of times had sexual intercourse in past for weeks | The bivariate analysis showed HIV/STI knowledge increases among female *ACES* participants (from M=3.69 at baseline to M=4.04 at 3-month-follow-up,  p=0.01, d=0.33, 95% CI=0.08–0.58), HIV/STI knowledge among *ACES* students 16 years of age or older (from M=3.86 at baseline to M=4.20 at 3-month follow-up,  p=0.03, d=0.28, 95% CI=0.027–0.52), favorable sexual values for male *ACES* participants (from M=3.57 at baseline to M=4.02 at 3-month follow-up,  p=0.01, d=0.33, 95% CI=0.08–0.58), and favorable sexual values for Latino male participants (from M=3.49 at baseline to M=3.93 at 3-month follow-up,  p=0.02, d=0.3, 95% CI=0.05–0.54). Among sexually initiated youth who at baseline reported having had sex three or more time in the past four weeks, *ACES* participants reported significant reductions in the number of times they had sex in the past four weeks (from M=13.3 at baseline to M=6.81 at 3-month follow-up, p=0.02, d=0.3, 95% CI=0.05–0.54). | Moderate |
| **“Risk avoidance”** (RA) and **“Risk reduction” (**RR)  Computer programs | Markham et al., 2012. | CRCT, EG1 (RA), EG2 (RR) & CG; United States; 7º, 8º, 9º graders; 15 middle schools. Pretest, 16-26-month-follow-up: 462 (EG1), 359 (EG2) and 435 (CG); 72.21%; 59.8%♀, 40.2% ♂; Mean age/SD: 12.6 (0.76); delayed sexual initiation, number of sexual relations in the last 3 months, unprotected sexual relations, number of sexual partners in the last 3 months, number of partners in the last 3 months, *Psychosocial*: STI/HIV knowledge, condoms; self-efficacy to expect or refuse sex, condom use; perceived beliefs about friends and sexual relationships; intention to have sex or not to have sex, condom use, STI testing; beliefs about condoms; communication with parents | Multivariate analysis of variance demonstrated efficacy in delaying sexual initiation for the *RA* and *RR* vs CG groups. In the EG *RA* for any sexual initiation the Hispanic group was less likely to initiate sex (AOR=0.40, 95% CI=0.19–0.86, p<0.05) than African-American group, no changes were observed according to sex. For oral sex, women were less likely to initiate sex (AOR=0.56, 95% CI=0.32–0.97, p<0.05), no change by race. For vaginal sex Hispanics were less likely to initiate (AOR=0.39, 95% CI=0.18–0.88, p<0.05), no changes by sex were observed. In the EG *RR* for any sexual initiation was less likely in general (AOR=0.65, 95% CI=0.54–0.77, p<0.01). It showed improvement in African American (AOR=0.38, 95% CI=0.18–0.79, p < 0.05) and women (AOR=0.43, 95% CI=0.31–0.60, p < 0.01). For oral sex women were less likely to initiate (AOR=0.44, 95% CI=0.26–0.75). For vaginal sex improved overall (AOR=0.64, 95% CI=0.45–0.93, p<0.05), in African Americans (AOR=0.32, 95% CI=0.15–0.67, p<0.01) and women (AOR=0.45, 95% CI=0.30–0.67, p<0.01). For the variable Unprotected sex at last vaginal intercourse, *RA* and *RR* were less likely to perform (*RA* AOR=0.70, 95% CI=0.52–0.93, p<0.05; *RR* AOR=0.67, 95% CI=0.47–0.96, p<0.05). To have sex in the next 3 months, were less likely *RR* group for vaginal and anal sex respectively (AOR=0.73, 95% CI=0.53–1.00, p<0.05 and AOR=0.53, 95% CI=0.33–0.84, p<0.01). Having sex without a condom was less likely in *RR* (AOR=0.59, 95% CI=0.36–0.95, p<0.05). | Moderate |
| ***“Teen Web”*** Web-based health education intervention**.** | Halpern et al., 2008**.** | Quasi-experimental, EG (x2) & CG (x2); 5 secondary schools in Kenya and 5 schools in Brazil. Pretest and immediate posttest: Kenya; 799 (EG), 379 (CG); 77%; 38.79%♀, 61.21%♂; Mean age/SD 16.50/019. Brazil; 559 (EG), 155 (CG); 51%; 54.2%♀, 45.8%♂; Mean age/SD 14.71/0.60; perceived barriers to condom (difficult to use, often break, too expensive, embarrassing to talk about, embarrassing to buy, embarrassing to use), condom use norms (only if many partners, even if trust partners, used by responsible partners, no condom, no sex, ok ask partner wear condom), condom effectiveness (pregnancy, HIV/AIDS), easy to obtain low cost condoms, easy to obtain HIV test, knowledge (What is emergency contraception (EC), hours EC effective, where to obtain EC, know abortion law) | Kenya: According to the controlled multivariate logistic regression analysis, *Web* students were more likely than comparison students to disagree that condoms ‘‘often break’’ (OR=1.64, 95% CI=1.07–2.54), but were less likely than comparisons to disagree that condoms are ‘‘too expensive’’ (OR=0.72, 95% CI=0.60–0.88) or ‘‘embarrassing to talk about’’ (OR=0.71, 95% CI=0.59–0.86). *Web* students were less likely than comparisons to endorse appropriate condom use when ‘‘one has many partners’’ (OR=0.71, 95% CI=0.62–0.81), when ‘‘one trusts their partner’’ (OR=0.87, 95% CI=0.81–0.93) and when “used by responsible partner” (OR=0.51, 95% CI=0.41–0.65). Students in web schools were less likely to perceive condoms as effective against HIV/AIDS (OR=6.58, 95% CI=2.68–16.15) and it’s easy for someone their age to get a condom at a low cost (OR=0.49, 95% IC=0.33–0.71). Students *web* and comparison schools did differ on knowledge about what emergency contraception (EC) is (OR=3.5, 95% CI=2.35–5.31), hours EC is effective (OR=2.99, 95% CI=2.60–3.45) and where to obtain it (OR=1.60, 95% CI=1.26–2.03). There was a web by gender interaction for perceptions about condom effectiveness in preventing HIV/AIDS; for girls, students in web schools were less likely to perceive condoms as effective against HIV/AIDS (OR=0.56, 95% CI=0.19–1.62). In contrast, boys in web schools were almost twice as likely as those in comparison schools to report condoms are effective against HIV/AIDS (OR=1.93, 95% CI=1.26–2.96). Also there were a web by gender interaction for students' perceptions about the difficulty of accessing condoms. For girls, students in web schools were four times more likely to agree that it is very or somewhat easy for someone their age to get a condom at a low cost (OR=4.60, 95% CI=2.56–8.24). Boys in web schools were also significantly more likely than those in comparison schools to agree with this statement, but the difference was smaller (OR=1.52, 95% CI=1.45–1.59).  Brazil: For statements reflecting perceived barriers to condom use, two showed main effects of intervention only. *Web* students were more likely to disagree that condoms often break (OR=1.44, 95% CI=1.06–1.95), but less likely to disagree with the statement that condoms are embarrassing to use (OR=0.44, 95% CI=0.26–0.74). *Web* students were less likely than comparisons to endorse appropriate condom use when ‘‘only if many partner’’ (OR=6.98, 95% CI=4.62–10.55) and when “used by responsible partner” (OR=0.41, 95% CI=0.36–0.47). Students in *web* schools were less likely to perceive condoms as effective against pregnancy (OR=0.10, 95% CI=0.03–0.33). *Web* students were much more likely to perceive easy access to low-cost condoms (OR=61.93, 95% CI=10.92–350.41). There was found a main effect of EG for ease of obtaining an HIV test (OR=1.80, 95% CI=1.48–2.18). The web intervention was associated with differences in knowledge about EC at posttest (OR=0.21, 95% CI=0.13–0.32), hours EC is effective (OR=2.25, 95% CI=1.61–3.14) and where to obtain EC (OR=0.34, 95% CI=0.25–0.45). Intervention effects varied by age for the statements “It is fine to say no to sex if there is no condom” and “It is all right for a woman to ask her partner to wear a condom.” (OR=0.67, 95% CI=0.50–0.90). In each case, web students were much more likely to endorse both statements, and differences were larger among younger students. Intervention effects differed by gender for only one statement, “Only people with many partners need to use condoms.” Girls in web schools were less likely than those in comparison schools to disagree with this statement (OR=0.14, 95% CI=0.03–0.58). There were no differences by web condition for boys (OR=1.00, 95% CI=0.45–2.26). | Strong |
| **Computer- and Internet-based intervention** | Roberto et al., 2008 | CRCT, EG & CG; United States; 10º grader; 2 schools. Pretest and posttest (3 weeks after the intervention/12 weeks from pretest): 147 (EG), 191 (CG); 89.45%; 56.7%♀, 43.3%♂ (EG); 45.9%♀, 54.1%♂ (CG); Mean age/SD (EG): 15.25/0.54, Mean age/SD (CG): 15.23/0.56; knowledge, susceptibility, condom efficacy, condom negotiation, situational efficacy, refusal efficacy, attitude toward waiting to have sex | A mixed-model two-way repeated-measures ANOVA, with treatment condition (experimental=control) as an independent groups factor, and time (pretest=posttest) as a within-subjects factor showed effect of the intervention on knowledge (M EG=8.47, M CG=6.35, d=0.90, 95% CI=0.67–1.13, p<0.001), condom negotiation (M EG=4.40, M CG=4.00, d=0.19, 95% CI=-0.02–0.41, p<0.05), condom efficacy (M EG=4.21, M CG=4.08, d=0.20, 95% CI=-0.02–0.41, p<0.05) and attitudes toward waiting to have sex (M EG=3.81, M CG=3.48, d=0.21, 95% CI=-0.01–0.43, p<0.05). | Moderate |
|  | Roberto, Zimmerman, Carlyle & Abner, 2007 | CRCT, EG & CG; United States; 10º grader; 2 schools. Pretest and posttest (approximately 10 weeks after the intervention finished): 139 (EG), 187 (CG); no data; 58.3%♀, 41.7%♂ (EG), 55.1%♀, 44.9%♂ (CG); Mean age/SD (EG): 15.50/0.63, Mean age/SD (CG): 15.68/0.73; knowledge, condom self-efficacy, attitude toward waiting, condom negotiation, situational self-efficacy, refusal self-efficacy, susceptibility (pregnancy), susceptibility (STDs), susceptibility (HIV), severity (pregnancy), , number partners in the last 4 months, initiation of sexual activity and condom use in the last intercourse (the two latter between those that were sexually active) | A two-way mixed-model repeated-measures analysis of variance with condition (treatment/control) as an independent groups factor and time (pretest/posttest) as a within-subjects factor showed effects of the program on knowledge (M EG=7.96, M CG=6.60, d=0.61, 95% CI= 0.39–0.83, p<0.001), condom negotiation (M EG=4.44, M CG=3.98, d=0.24, 95% CI=0.02–0.46, p<0.05), attitudes towards waiting to have sex (M EG=3.71, M CG=3.44, d=0.19, 95%CI=-0.03–0.41, p<0.05,), situational self-efficacy (M EG=4.05, M CG=3.64, d=0.23, 95% CI=0.006–0.45, p<0.05) and susceptibility (M EG=1.63, M CG=1.86, d=0.20, 95% CI=-0.02–0.42, p<0.01).  Logistic regression was used to assess the impact of the intervention on initiation of sexual activity using only individuals who were not already sexually active at Time 1 (231 subjects [74.4% EG; 72.9% CG]), with a main effect for condition (p < .01, 33 (18%) students in the CG initiated sexual activity between the pretest and the post-test, and 10 (8%) in the EG, OR = 2.93). | Moderate |
|  | Roberto, Zimmerman, Carlyle, Abner, et al., 2007 | Quasi-experimental research, EG & CG; no data; 9º grader; 9 schools. Pretest and posttest: 550 (EG), 337 (CG); no data; 54.9%♀, 45.1%♂ (EG), 51.9%♀, 48.1%♂ (CG); Mean age/SD (EG): 14.49/0.63, Mean age/SD (CG): 14, 37/0,58; knowledge, condom self-efficacy attitude toward waiting, condom negotiation, situational self-efficacy, refusal self-efficacy, susceptibility (pregnancy), susceptibility (STDs), susceptibility (HIV), severity (pregnancy) | A two-way mixed-model repeated-measures analysis of variance with condition (treatment/control) as an independent groups factor and time (pretest/posttest) as a within-subjects factor showed effects of the program on knowledge (M EG=4.29, M CG=4.10, p<0.05, d=0.13, 95%CI=-0.007–0.26), condom self-efficacy (M EG=4.25, M CG=4.23, p<0.01, d=0.19, 95% CI=0.05–0.32), attitudes towards waiting to have sex (M EG=3.42, M CG=3.35, p=0.05, d=0.11, 95% CI=-0.02–0.25) and perceived increased susceptibility on HIV (M EG=1.82, M CG=1.63, p<0.02, d=0.16, 95% CI=0.03–­0.3). | Strong |
| **“CAI”** Computer-assisted instruction: | Kann, 1987 | Quasi-experimental, EG & 2 CG; United States; 7º-12º graders; 4 secondary schools. Pretest, posttest and delayed posttest (5 weeks after posttest): 151 (EG: CAI), 147 (CG1: RCI regular classroom instruction), 93 (CG2: no intervention); 65%; 51.4% ♀, 48.6% ♂; Age: 12-19; decision-making behavior, assertiveness behavior, interpersonal communication behavior, interpersonal communication attitude, assertiveness attitude, decision making knowledge, assertiveness knowledge and interpersonal communication knowledge | Split-plot factorial analysis was conducted on each dependent variable for all subjects combined. Subsequent one-way analysis of variance (ANOVA) and Scheffe’ tests determined specific sources of difference between the EG and CG. Decision-making behavior (EG pre=30.86, EG post=32.19, EG delayed=31.46, EG F=0.044, p<0.05; CG1 pre=30.86, CG1 post=31.98, CG1 delayed=30.25, CG1 F=0.057, CG2 pre=29.91, CG2 post=30.69, CG 2 delayed=31.77, CG2 F=0.274 ), assertiveness behavior (EG pre=12.85, EG post=14.37, EG delayes=13.04, EG F= 0.041, p<0.05; CG1 pre=13.61, CG1 post=13.61, CG1 delayed=13.68, CG1 F=0.995; CG2 pre=11.98, CG2 post=12.55, CG2 delayed=13.23, CG2 F=0.535), Interpersonal communication behavior (EG pre=29.74, EG post=30.93, EG delayed=31.31, EG F=0.047, p<0.05; CG1 pre=29.59, CG1 post=29.78, CG1 delayed=29.04, CG1 F=0.908; CG2 pre=30.30, CG1 post=30.03, CG2 delayed=30.35, CG2 F=0.930), interpersonal communication attitude (EG pre=22.72, EG post=29.44, EG delayed=28.40, EG F=0.043, p<0.05; CG1 pre=27.72, CG1 post=28.02, CG1 delayed=28.38, CG1 F=0.532; CG2 pre=25.62, CG2 post=27.33, CG2 delayed=26.53, CG2 F=0.446), decision making knowledge (EG pre=2.59, EG post=2.89, EG delayed=2.60. EG F=0.021, p<0.05; CG1 pre=2.39, CG1 post=2.56, CG1 delayed=2.52, CG1 F=0.388; CG2 pre=2.45, CG2 post=2.52, CG2 delayed=2.22, Cg2 F=0.188), assertiveness knowledge (EG pre=2.40, EG post=2.74, EG delayed=2.44, EG F=0.012, p<0.05; CG1 pre=2.28, CG1 post=2.49, CG1 delayed=2.17, CG1 F= 0.051, CG2 pre=2.30, CG2 post=2.10, CG2 delayed=1.98, CG2 F=0.134) and interpersonal communication knowledge (EG pre=1.65, EG post=1.91, EG delayed=1.68, EG F=0.037, p<0.05; CG1 pre=1.72, CG1 post=1.84, CG1 delayed=1.73, CG1 F=0.060; CG2 pre=1.56, CG2 post=1.56, CG2 delayed=1.42, CG2 F=0.506) improved significantly following CAI but no after RCI. The impact of CAI decayed significantly between the posttest and delayed test for the knowledge variables, but not for the attitude and behavior variables.  When the data were analyzed according to gender, CAI significantly improved decision-making knowledge, assertiveness knowledge and behavior, and interpersonal communication knowledge for females. However, for males, CAI significantly improved decision-making knowledge and behavior, assertiveness knowledge and behavior, and interpersonal communication attitude and behavior (data not shown).  For seventh and eighth grades, CAI significantly improved decision-making knowledge and behavior and assertiveness behavior. For ninth and tenth grades, CAI significantly improved decision-making behavior, assertiveness behavior, and interpersonal communication knowledge, attitude, and behavior. For 1lth and 12th grades, CAI significantly improved decision-making knowledge and behavior, assertiveness knowledge and behavior, and interpersonal communication knowledge and behavior (data not shown).  Analysis of data according to school-community setting indicated CAI significantly improved assertiveness behavior and interpersonal communication knowledge and attitude at both urban schools. At one rural school, assertiveness knowledge and interpersonal communication knowledge improved significantly from CAI, while at the other rural school, CAI significantly improved decision-making knowledge and behavior and assertiveness behavior (data not shown). | Moderate |

CRCT: cluster randomized clinical trial; EG: Experimental Group; CG: Control Group; SD: Standard Deviation; RCI: regular classroom instruction; SRH: sexual and reproductive health; SG: serious game; GM: gamification GLM: generalized linear model

**Table S3. *Intervention's characteristics***

| **Project** | **Objective** | **Target population; Setting; Course** | **Facilitators** | **Intervention characteristics; Material and resources; Theory** | **Number of sessions; Session frequency; Duration** | **Intervention content** |
| --- | --- | --- | --- | --- | --- | --- |
| **“You and Me”:** Internet-based sexuality education program  (Hu et al., 2023; Jin et al., 2021) | To improve sexual knowledge, attitudes, and behaviors among adolescents | General adolescents;  High school; 10º grader. | Teachers (previously trained) are only required to facilitate the implementation of the contents | Online sessions designed by sexual and reproductive health experts. Each session introduces one sex-related topic; Videos, PowerPoint slides and predesigned class activities; No data | 8 sessions; 1 per week; 45 minutes | - sexuality education - gender the reproductive system - puberty pregnancy and contraception - disease and behavior - sexual violence - relationship and marriage |
| **“Media Aware”:** Mobile health (mHealth), media literacy education program  (Scull et al., 2022; Scull et al., 2018) | To improve adolescent critical thinking about media messages and provide medically-accurate information and skills building related to sexual health and communication | General adolescents; School; 7º, 8º, 9º, 10º graders. | Teachers (previously trained) are required to use a script to distribute consent and assent form packets to students in participating classrooms | Each lesson contains two or three smaller mini-lessons; No data; Elaboration Likelihood Model, Heuristic-Systematic Model, Message Interpretation Process model, Theories of Reasoned Action and Planned Behavior | 4 sessions; No data; 45 minutes | - Pregnancy and contraception - Sexually Transmitted Infections - Media literacy education (MLE) |
| **“HEART”:** Interactive online program  (Widman et al, 2020; Widman et al, 2018) | To prevent and reduce risk sexual behaviors | General adolescents (Widman et al., 2020) and rural, low-income high schools (Widman et al., 2018); School; 9º, 10º graders | No data | An interactive, skills-focused intervention designed for digital delivery in one sitting; Audio/video clips, tips from other teens, interactive games/quizzes, infographics, and skill-building exercises with self-feedback given in real-time; Reasoned Action Model and Fuzzy Trace Theory | 1 session; No data; 45 minutes | - safer sex motivation - HIV/STD knowledge - sexual norms/attitudes - safer sex self-efficacy - sexual communication skills |
| ***“*Cuídalos”:** Web-based intervention (Varas-Díaz et al., 2019) | To increase sexual communication between parents and adolescents | Latino adolescents and their parents; Community organization and public school; No data | Project team | The parents carried out 6 modules with interactive activities where they learn how to relate to their children, where they are taught possible reactions of their children and how to act. They had a homework to do with their children. ; Animation, videos…; Ecodevelopmental Theory, Theory of Reasoned Action/Planned Behavior, and Social Cognitive Theory | 6 parents modules; in two days; 60 minutes | - Knowledge about pregnancy, HIV/AIDS and STDs - Support attitudes and develop skills to facilitate communication - HIV/AIDS stigma |
| **“Pathways for African American Success” (PAAS)):** Technology-delivered [HIV risk](https://www--sciencedirect--com.us.debiblio.com/topics/medicine-and-dentistry/human-immunodeficiency-virus-risk-behavior) prevention program (Murry et al., 2019; Murry et al., 2018) | To prevent HIV-related risk behavior by harnessing protective parenting processes | Rural adolescents and their parents; School; 6º grader | Technology Intervention Assistants (previously trained) in self-directed technology condition: directed participants to an available laptop, provided any necessary technical assistance, and remained in viewing sight to oversee session completion | A combination of parent sessions, youth sessions, and family sessions (with parent–child pairs). PAAS is a Self-directed technology condition, Facilitator-led small group condition is role-playing activities and guided discussions with time allotted to ask questions and Home mailed literature control condition; No data; Cognitionism, constructionism and behaviorism | 6 parent sessions, 6 youth sessions, 6 family sessions; 1 per week, 90 minutes | - Supportive parenting - Establishing family rules and routines - Nurturing involved parenting - Adaptive racial socialization and encouraging racial pride - Linking school and academic performance to goals, dreams to youths’ future orientation - Protecting against dangerous behavior - Parental protections that reduce high-risk behaviors |
| ***“*It's Your Game” (IYG):** Computer program(Peskin et al., 2019; Potter et al., 2016; Tortolero et al., 2010) | To prevent HIV, sexually transmitted infection (STI), pregnancy and to delay sexual initiation. | Low-income adolescents; Middle school; 7º, 8º graders | Facilitator trained | ; Group-based classroom activities with personal journaling and individual activities delivered on laptop computer; Intervention Mapping: social cognitive theory, social influence models and the theory of triadic influence. | 24 sessions; Twelve 7th-grade and twelve 8th-grade 45-minute lessons; 45 minutes | - Characteristics of healthy friendships - Setting personal limits and practicing refusal skills in a general context - Information about puberty, reproduction and STIs - Characteristics of healthy dating relationships - HIV, STI, pregnancy testing, condom and contraceptive use. |
| **“It's Your Game (IYG)-Tech”:**  Computer program  (Peskin et al., 2015) | To prevent HIV, sexually transmitted infection (STI), pregnancy and to delay sexual initiation. | General adolescents; School; 8º grader | Standardized data collection procedures were implemented in all survey sites by trained data collectors | The curriculum is set within a mall-like environment that includes several “storefronts” and “proprietors”; Animated scenarios with modeling and skills practice, peer modeling videos, quizzes, fact sheets, a graffiti wall, virtual role-play activities; Life-skills paradigm: Select, Detect, Protect | 13 sessions; No data; 35-45 minutes | - Characteristics of healthy and unhealthy friendships and dating relationships - Anatomy and reproduction - Social, emotional, and physical consequences of sex - Communication skills - Teen pregnancy and STIs - Knowledge and skills for condom and contraception use - Condom negotiation |
| **“Malaysian Care for Adolescent Project” (MyCAP):** Website for online SRH education  (Nik Farid et al., 2018) | To improve knowledge about sexual and reproductive health | Low-to middle-class community; School; No data | No data | A short introduction and one educational session consisting of 17 SRH topics and 1.5 hour to browse the website; Videos, informational graphs and articles; Precede-Proceed model | 1 session; No data; 90 minutes | - Reproductive organs - Puberty - Menstruation - Wet dreams - Masturbation - Sexual health - Sexual feeling - Teenage pregnancy - Pornography - Dating - Premarital sex - Contraceptive methods - STI - Abortion - Abandoned babies - Sexual abuse - Attitude towards SRH |
| **Smartphone app**  Jeong et al., 2017 | To improve sexual health knowledge | General adolescents; High school; 3º grader | Research assistants (previously trained) were collected data | 30 minutes self-study and students should commit to allocating 15 minutes per week to self-study for 5 weeks using the educational booklet or smartphone application; Website, cartoon clips; Roger’s protection-motivation theory | 5 sessions; 1 per week; 45 minutes | - STI risks - STI knowledge - STI prevention skills - STI coping skills and websites related to STIs on the Internet |
| **Website**  Doubova et al, 2017 | To prevent and reduce risk sexual behaviors | Socioeconomically disadvantaged neighborhoods; School; 3º year of secondary school | A psychologist member of the research team conducted sessions face-to-face. Also, teachers motivated students through in-class reminders, emphasizing the importance of their participation | A website that included educational sessions; Comics, games, role-playing; The information−motivation−behavioral skills model | 4 web sessions and 6 face-to-face sessions; per week (web) and for 3 months (face-to-face); 60 minutes (web) and 30 minutes (face-to-face) | - Dating - Courtship - Sexual relationship - Clarification of misconceptions and myths about gender-roles and sexual relationships - Partner abuse - STIs - Early pregnancy - Self-esteem - Use of male and female condoms and examples for condom negotiation |
| **“PlayForward”:** Video game (Fiellin et al., 2017; Fiellin et al., 2016) | To improve adolescent sexual health outcomes | Minority adolescents; Urban after-school, school, and summer camp program; No data | Research personnel observed and documented both the PlayForward and the control group participants’ duration of gameplay | PlayForward is a two-dimensional role-playing adventure video game that focuses primarily on individual decision making.; Mini games; Theoretical base | 12 sessions; twice a week; 60 minutes | - Sexual health - Risky behaviors: substance use, academic dishonesty and unsafe driving |
| **“Connect”:** Internet-based intervention: (Castillo-Arcos et al., 2016) | To reduce sexual risk behaviors and increase resilience to sexual risk behaviors | Mexican adolescents; Urban educational; No data | Facilitators from the health field, each of whom delivered the intervention to 10–13 participants selected at random | ; No data; Conceptual framework of Adolescent Sexual Resilience | 6 online sessions and 2 face-to-face sessions (sessions 1 and 8); No data; 60 minutes | - Information about HIV/AIDS - Identifying protective factors: family, social and/or individual - Strengthening levels of self-esteem and self-efficacy - Social skills and effective communication - Resilience |
| **“CyberSenga”:** Healthy sexuality program (Ybarra et al., 2015; Ybarra et al., 2013) | To prevent HIV | Ugandan adolescents; School; 9º, 10º, 11º graders | Research assistants directed participants to a computer and helped them login to the CyberSenga system as needed and provided appointment reminder cards and actively sought out participants who did not show up for their scheduled sessions so that they could complete the module the same day or reschedule for the next day | ; Vignettes, videos…; Information-Motivation-Behavioral Skills Model | 5 sessions and 1 review session; per week; 45 minutes | - Information and facts about HIV - Motivation by focusing on problem-solving and communication skills - Motivations to have sex or, alternatively, to be abstinent - Behavioral Skills - Culturally-specific issues related to HIV preventive behavior |
| ***“*Abstinence and Contraception Education Storehous” (ACES):**  Digital library (Raghupathy et al., 2013) | To reduce sexual risk behavior | General adolescents; Secondary school and a Planned Parenthood service: No data | Science teachers explained the study design and process to parents and students, procedures for participation, and obtained parental consent and student assent forms | ; Audio-visual, presentations, video clips, quizzes and polls, role-play, exercises for modeling and rehearsal, group discussion…; No data | 6 sessions; in 2 weeks; 55 minutes (2 sessions) and 2 hours (4 sessions) | - HIV/AIDS awareness: “How do you get HIV/AIDS”, - Condom demonstration, - Negotiation skills: “I want him to wear a condom” - Buying contraception; “A trip to the drug store”). |
| **“Risk avoidance”** (RA) and **“Risk reduction”** (RR): Computer program (Markham et al., 2012) | To delay sexual initiation | African-American or Hispanic adolescents; Middle school: 7º,8º,9º graders | Facilitators received a 5-day training, which modeled RA or RR lessons by skilled trainers and provided teaching practice. Facilitators received technical support throughout implementation. | . Each program integrated group-based classroom activities with individual journaling and computer-based activities; Activities in the classroom with personalized journaling and individual, tailored, computer-based activities and 3 parent-child activities to facilitate dialogue on topics including friendship qualities, dating, and sexual behavior; Intervention Mapping (IM): social cognitive theory, social influence models and the theory of triadic influence | 24 sessions; twelve lessons in 7º grade and twelve lessons in 8º grade; 50 minutes | **Risk avoidance**   - Beliefs about the benefits of abstinence until marriage and the benefits of marriage and parenting within marriage - Character qualities and their influence on healthy relationships and decision making.   **Risk reduction**   - Beliefs about the benefits of abstinence until old age - Self-respect and responsibility - Knowledge and self-efficacy of condom and contraceptive use |
| **“Teen Web”:** Web-based health education intervention (Halpern et al., 2008) | To improve knowledge and attitudes about topics such as condom use, access to HIV testing, emergency contraception and abortion laws | Adolescents of low resource settings; School; No data; | No data | Complete one web-based sexual health module approximately every 6–8 weeks, and in return, had free access to the Internet for at least 30 min after completing each module; No data; No data | 6 sessions; per six-eight weeks; No data | - Substance use - Sexuality - Contraception - Voluntary HIV counseling and testing - Abortion law - Intimate partner violence |
| **Computer and Internet based intervention** (Roberto et al., 2008; Roberto, Zimmerman, Carlyle & Abner, 2007, Roberto, Zimmerman, Carlyle, Abner, et al., 2007) | To prevent pregnancy, STD and HIV | Rural adolescents; School; 10º grader | Researchers and technical support staff closely monitored program implementation throughout the intervention | Included six computer-based activities. One activity was put online for each of the first 6 weeks, and all six activities were put online during week seven to encourage and enhance participation; Sensation-Seeking Scale, Impulsive Decision-Making Scale, CD-ROM, Radio PSA Contest; Extended parallel process model | 7 sessions; 1 per week; 15 minutes | - Sexual health - Health behavior - Refusal skill |
| **“CAI”:** Computer-assisted instruction (Kann, 1987) | To improve decision-making, assertiveness and interpersonal communication related to responsible sexuality | Secondary school; 7º, 8º, 9º, 10º, 11º, 12º graders | Instructor (previously trained) are only required to facilitate the implementation of the contents | Three original simulation-based programs which began with background material on interaction skills followed by directions for completing upcoming exercises; No data; No data | 3 sessions; No data; 30 minutes | No data |
